# Comparative whole genome DNA methylation profiling of cattle sperm and somatic tissues reveals striking hypomethylated patterns in sperm

**DOI:** 10.1093/gigascience/giy039

**Published:** 2018-04-10

**Authors:** Yang Zhou, Erin E Connor, Derek M Bickhart, Congjun Li, Ransom L Baldwin, Steven G Schroeder, Benjamin D Rosen, Liguo Yang, Curtis P Van Tassell, George E Liu

**Affiliations:** 1 Key Laboratory of Agricultural Animal Genetics, Breeding and Reproduction, Education Ministry of China, Huazhong Agricultural University, Wuhan, Hubei, 430070, China; 2 Animal Genomics and Improvement Laboratory, BARC, US Department of Agriculture, Agriculture Research Service, Beltsville, MD 20705, USA; 3 The Cell Wall Utilization and Biology Laboratory, US Department of Agriculture, Agriculture Research Service, Madison, WI, 53706, USA

**Keywords:** cattle, sperm, somatic cells, DNA methylation, hypomethylated region, WGBS (whole genome bisulfite sequencing)

## Abstract

**Background:**

Although sperm DNA methylation has been studied in humans and other species, its status in cattle is largely unknown.

**Results:**

Using whole-genome bisulfite sequencing (WGBS), we profiled the DNA methylome of cattle sperm through comparison with three somatic tissues (mammary gland, brain, and blood). Large differences between cattle sperm and somatic cells were observed in the methylation patterns of global CpGs, pericentromeric satellites, partially methylated domains (PMDs), hypomethylated regions (HMRs), and common repeats. As expected, we observed low methylation in the promoter regions and high methylation in the bodies of active genes. We detected selective hypomethylation of megabase domains of centromeric satellite clusters, which may be related to chromosome segregation during meiosis and their rapid transcriptional activation upon fertilization. We found more PMDs in sperm cells than in somatic cells and identified meiosis-related genes such as*KIF2B* and *REPIN1*, which are hypomethylated in sperm but hypermethylated in somatic cells. In addition to the common HMRs around gene promoters, which showed substantial differences between sperm and somatic cells, the sperm-specific HMRs also targeted to distinct spermatogenesis-related genes, including *BOLL*, *MAEL*, *ASZ1*, *SYCP3*, *CTCFL*, *MND1*, *SPATA22*, *PLD6*, *DDX4*, *RBBP8*, *FKBP6*, and *SYCE1*. Although common repeats were heavily methylated in both sperm and somatic cells, some young Bov-A2 repeats, which belong to the SINE family, were hypomethylated in sperm and could affect the promoter structures by introducing new regulatory elements.

**Conclusions:**

Our study provides a comprehensive resource for bovine sperm epigenomic research and enables new discoveries about DNA methylation and its role in male fertility.

## Background

DNA methylation plays important roles in normal development and is associated with many processes such as gene expression, genomic imprinting, repression of transposable elements, and gametogenesis [1–5]. DNA methylation changes dramatically during mammalian development, and aberrant methylation patterns may lead to numerous diseases [6, 7]. Compared to somatic cells, sperm cells undergo nearly complete reprogramming of DNA methylation and exchange histones by protamine [8–14]. Sperm DNA methylation patterns have been well characterized in a few species, including humans and rodents [15–20]. These studies found that proper DNA methylation in sperm is required for successful meiosis [21]. In humans, sperm DNA has eight times more hypomethylated loci than DNA from other somatic cells [14, 22]. Additionally, sperm DNA hypermethylation has been associated with poor sperm parameters, idiopathic male infertility, and even pregnancy failure [23–29].

Transposable elements or common repeats constitute roughly half of most mammalian genomes [30]. Repression of these common repeats relies on DNA methylation via the piRNA pathway and is essential for the maintenance of genomic stability in the long term and for germ cell function in the short term [31, 32]. In humans, common repeats were found to be heavily methylated, with the notable exclusion of young AluY and AluYa5 elements in human sperm cells [33]. If methylation is lost on certain repressed repeats, germ cell development is arrested in meiosis.

Our knowledge of DNA methylation patterns in livestock is still limited when compared to humans and other model species. A few DNA methylation studies were reported with limited tissue types and low resolution in cattle, pigs, sheep, and horses [34–48]. As the species benefitting most from artificial insemination, we aimed to profile the cattle sperm DNA methylome through comparison with somatic cells from three tissues (mammary gland, brain prefrontal cortex, and blood). We constructed their DNA methylation profiles using the whole-genome bisulfite sequencing (WGBS) method. We investigated the landscapes of the DNA methylome in sperm as compared to the somatic cells. We studied differential methylation by comparing them in multiple contexts, including global CpGs, pericentromeric satellites, partially methylated domains (PMDs), hypomethylated regions (HMRs), and common repeats. In line with the Functional Annotation of Animal Genome project [49], this study provides a comprehensive resource for bovine sperm epigenomic research and enables new discoveries about DNA methylation and its role in male fertility.

## Results

### Methylomes of sperm and somatic tissues in cattle

We generated single-nucleotide resolution methylation profiles of sperm and somatic cells from three tissues from cattle. The somatic tissues were mammary gland, blood, and brain prefrontal cortex collected from two cows as biological replicates. Semen was collected twice for each of two bulls, respectively. Through WGBS, we obtained considerable data; 2.1 billion unique mapped reads for the three somatic cell types and 1.3 billion unique mapped reads for sperm (Table 1). For each of the 10 samples, 85.5 to 95.6% of the whole cattle genomic CpGs were covered with the average depth from 5.5 to 7.2 × (Table 1). Across the whole genome, CpG dinucleotides were preferentially methylated. Genome wide, we saw a CpG methylation rate of 72.8 to 78.1% across all samples (Table 1). Bisulfite conversion rates estimated by unmethylated lambda DNA controls supported that we faithfully captured patterns of genomic DNA methylation in these samples (Table 1). Moreover, we detected less than 0.8% non-CG methylation in the non-brain somatic tissues (mammary gland and blood) and sperm cells in contrast to a higher (∼1.3%) non-CG methylation level in the brain samples, which is consistent with previous studies in other species [50].

**Table 1:** Whole genome bisulfite sequencing of sperm cells (Sperm) and somatic cells from brain prefrontal cortex (CORTEX), mammary gland (MAM), and blood (WBC) of dairy cattle

Sample	Mapped reads	Bisulfite conversion rate (%)	Methylation level (%)	Average CpG coverage (× fold)	Whole genome CpG covered (%)
CORTEX1	407,336,580	99.31–99.44	74.50	7.03	95
CORTEX2	437,071,564	99.34–99.47	76.30	6.39	86
MAM1	344,055,380	99.38–99.48	73.10	6.19	94
MAM2	393,720,380	99.38–99.44	72.80	7.18	96
WBC1	280,887,870	99.40–99.46	78.10	6.41	94
WBC2	282,543,428	99.25–99.44	77.50	6.83	93
Sperm1 A	296,241,574	99.43–99.52	74.00	5.75	95
Sperm1 B	302,185,176	99.43–99.51	75.70	5.49	93
Sperm2 A	368,996,008	99.40–99.51	76.40	7.10	96
Sperm2 B	289,192,004	99.34–99.41	75.70	5.75	94

### Global comparisons between sperm methylomes and somatic tissue methylomes

**Figure 1:**
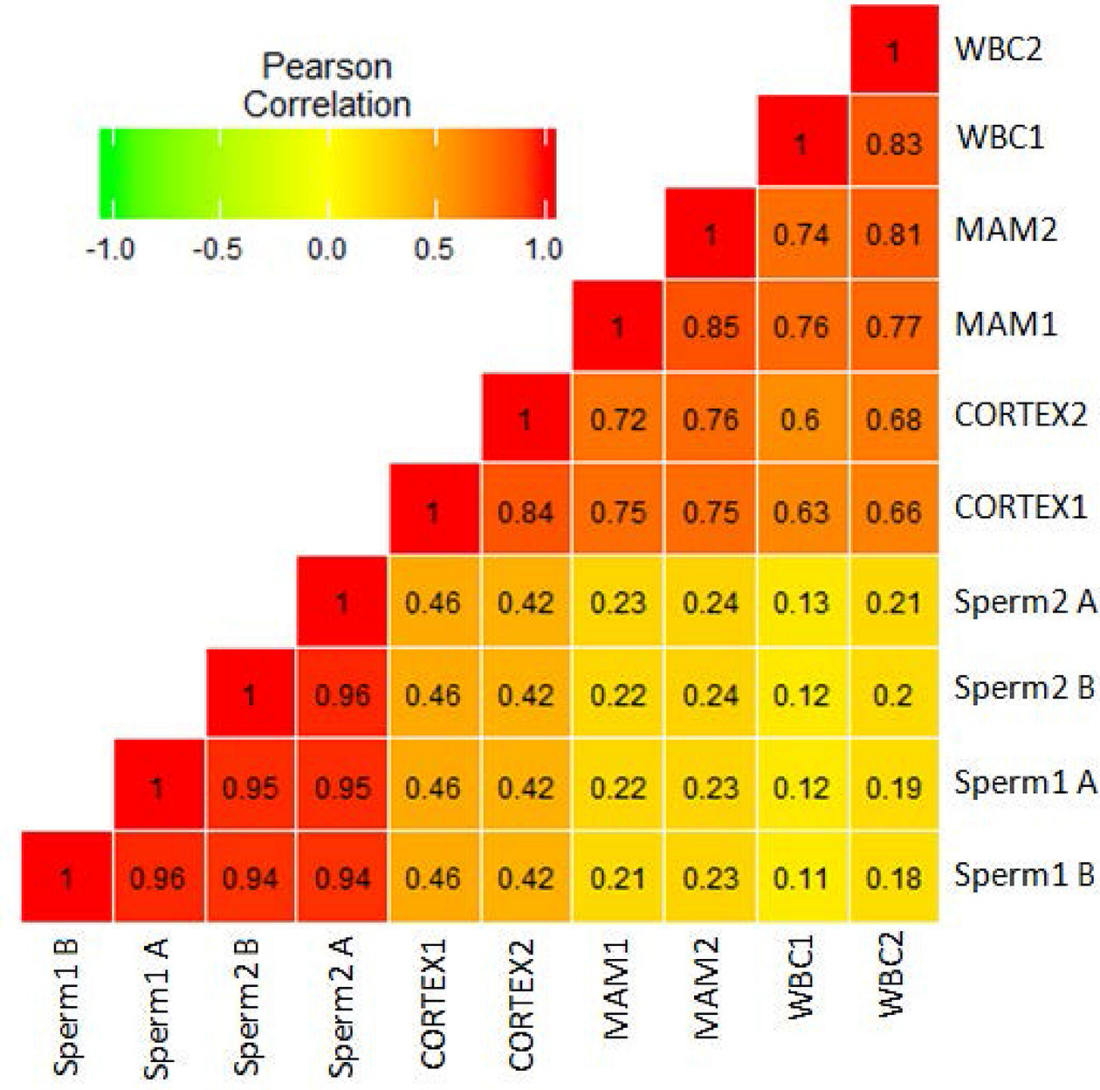
Correlation analysis between each sample using common CpGs. Sperm1 A and B: sperm samples from Holstein 1; Sperm2 A and B: sperm samples from Holstein 2; WBC: whole blood cells; MAM: mammary glands; CORTEX: prefrontal cortex of the brain.

We compared the methylation profiles between pairs of samples at a global CpG level. As expected, the correlations between samples within the same tissue or within sperm were high (*r* > 0.8) (Fig. 1). The correlations between methylation of different tissues were lower, especially the correlation efficiency between sperm and somatic cell methylation, which ranged from 0.11 to 0.46 (Fig. 1). Cluster analysis according to the CpG methylation also confirmed the consistent results of the biological replicates and reinforced potential methylation differences between somatic cells and sperm cells (Supplementary Fig. S1). PC1 of the principal component analysis (PCA) explained most of the variances and successfully separated sperm cells from somatic cells (Supplementary Fig. S2). PC2 of the PCA explained most of the variances within somatic cells and successfully separated brain from the other somatic tissues (Supplementary Fig. S2). Moreover, we detected 73,023 differentially methylated cytosine (DMCs) in autosomes between sperm cells and somatic cells (Supplementary Table S1). These results indicate large differences between sperm and somatic cell methylomes, possibly related to sperm development, in which the genome undergoes a wave of nearly complete demethylation and remethylation.

Next, we performed a global comparison of distinct genomic features between cattle sperm cells and somatic cells. Both cell types showed high methylation levels for the genic and most of the common repeats and showed comparably low methylation levels for CGI, promoters, low complexity sequence, and tRNA (Supplementary Fig. S3). The satellite was the most variable with significantly lower methylated genome features (*p* < 0.01) in sperm than that in somatic tissues (Supplementary Fig. S3). In contrast, similar methylation levels were seen for all other genomic features between sperm cells and somatic cells. Most of the methylation levels of genomic features showed unimodal patterns of either high or low. Promoter and CGI showed obvious bimodal patterns, which supports their functions in the regulation of gene expression. We also found parts of promoter and CGI with obviously different methylation levels between sperm and somatic cells (Supplementary Fig. S4). Apart from those, the satellites had largely low to medium methylation levels in sperm cells. Furthermore, the satellites showed globally different methylation patterns between brain (enriched in medium methylation) and the other two somatic tissues (high methylation) (Supplementary Fig. S4).

### Different methylation patterns in the partially methylated domains between sperm and somatic cells

To get exact knowledge of the methylation differences between somatic cells and sperm cells, we binned the cattle genome into nonoverlapping 20-kb windows. The methylation level of 20-kb windows in sperm was mainly enriched at 80%–100%;in somatic cells, the methylation level distributed more dispersedly and was enriched at 60%–100% (Supplementary Fig. S5a). Although there was no clear indication for bimodal distribution in both somatic and sperm cells, sperm exhibited significantly (*p* < 0.01) more low methylated windows than somatic tissues (∼3% vs. 1.2%) when limiting the average methylation level to <50% (Supplementary Fig. S5b, S5c). Moreover, at the chromosome level, obviously more PMDs were seen in the sperm cells than in the somatic cells (Supplementary Fig. S6), e.g., chr7, chr15, chr18, chr21, chr23, and chr29. We identified 69 contiguous PMDs that were 47 Mb in length for sperm cells using a hidden Markov model, among which 37 PMDs were supported by at least one kind of somatic tissue (Supplementary Table S2). However, all 37 PMDs were derived from brain, and only 3 PMDs were from blood samples (Supplementary Table S2).

**Figure 2:**
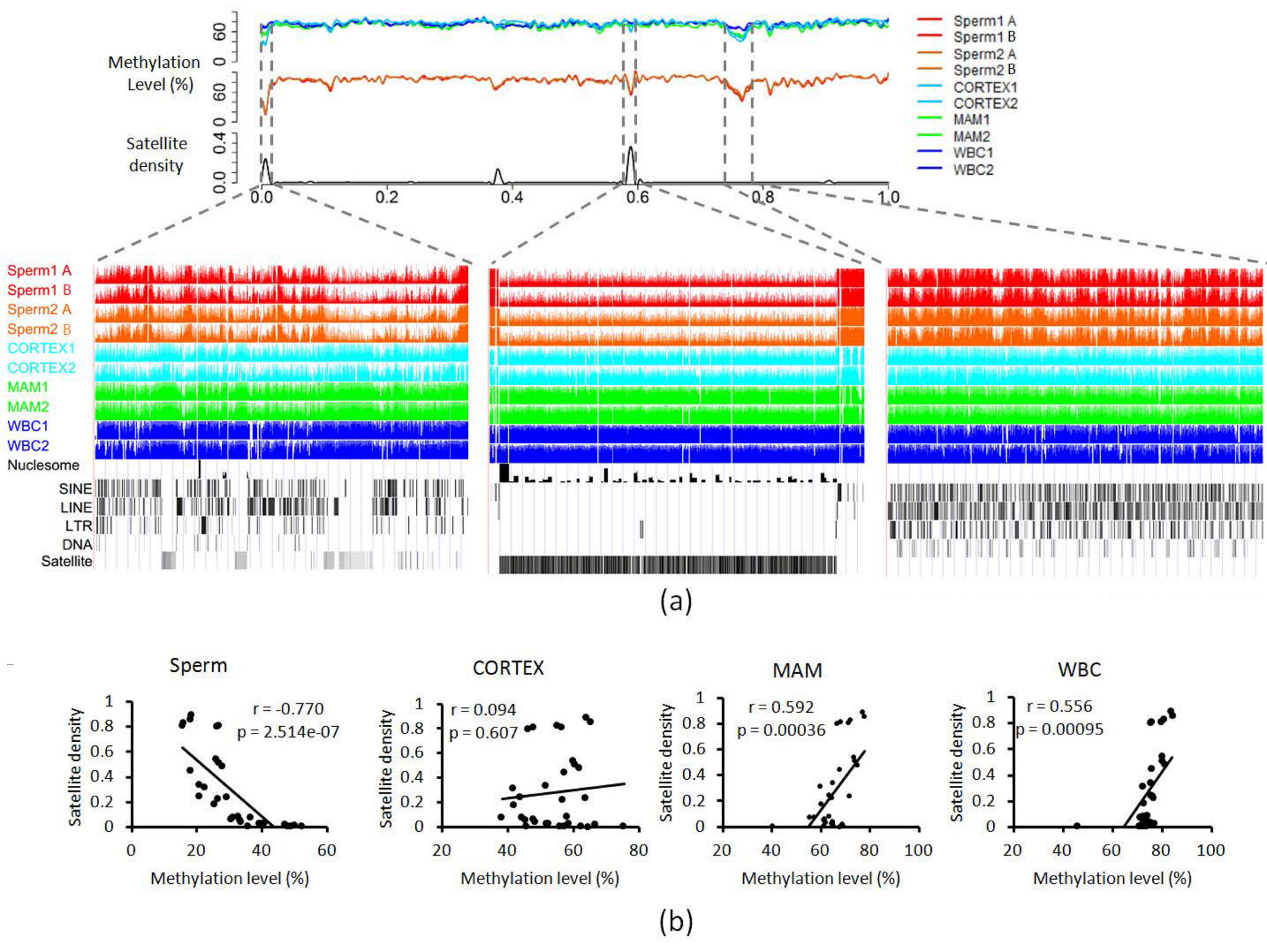
Characteristics of the sperm cell PMDs. **(a)** CpG methylation status of the PMDs using chr29 as an example. PMDs are indicated by the dashed lines. **(b)** Correlation analysis between satellite density and methylation levels of PMDs in sperm cells and somatic cells. Sperm1 A and B: sperm samples from Holstein 1; Sperm2 A and B: sperm samples from Holstein 2; WBC: whole blood cells; MAM: mammary glands; CORTEX: prefrontal cortex of the brain.

We evaluated the enrichment of different genomic features by calculating the ratio (observed/expected [O/E]) between the observed density in sperm-specific PMDs and the average density in autosomes (Supplementary Fig. S7). The PMD contained fewer genic regions (O/E = 0.36), more CGI (O/E = 1.74),and more satellite regions, which received the highest O/E value of 21.31. A previous study identified that the satellite enriched pericentromeric regions showed strongly decreased methylation in human sperm but not in human embryonic stem cells [14]. The localizations of functional bovine pericentromeres are currently unknown but estimated to be near the start of the chromosomes (Supplementary Fig. S6 and Supplementary Table S2). In our study, we observed clear PMD enrichment (20/69 within the first 3 Mb or 35/69 in the first 10% terminal regions of the chromosomes) in cattle sperm cells. Although a few of the PMDs starting from the chromosome start sites were also observed in somatic cells, the interstitial satellite regions showed strongly decreased methylation in sperm cells when compared to somatic cells (Supplementary Fig. 2a, left panel; chr29:1–560,000). In the middle of the chromosome, lowly methylated satellite regions contributed to some of the sperm-specific PMDs (Fig. 2a, middle panel; chr29:30,220,001–30,400,000). The 32 satellite-containing PMDs showed lower methylation levels than the nonsatellite-containing PMDs in sperm, which was not seen in the somatic tissues (Supplementary Fig. S8). Moreover, significantly negative correlation (*r* = –0.77, *P* = 2.514e-07) between satellite densities (i.e., total satellite length divided by the region length; Supplementary Table S2) and methylation levels were seen in sperm cells (Fig. 2b). Among somatic tissues, both mammary gland and blood showed significantly positive correlation (*r* = 0.59, *P* = 0.00036; *r* = 0.56, *P* = 0.00095) between satellite densities and methylation levels, while the brain showed no significant correlation (*P* = 0.61) (Fig. 2b). Additionally, different methylation patterns in the PMDs appeared in both sperm and somatic cells. For example, the PMD located in the chr29:38860,001–39,780,000 region showed multiple discontinuously HMRs in sperm cells, which was not seen in cells from mammary gland or brain (Fig. 2a, right panel).

### Function analysis of the genes located in sperm PMDs

**Figure 3:**
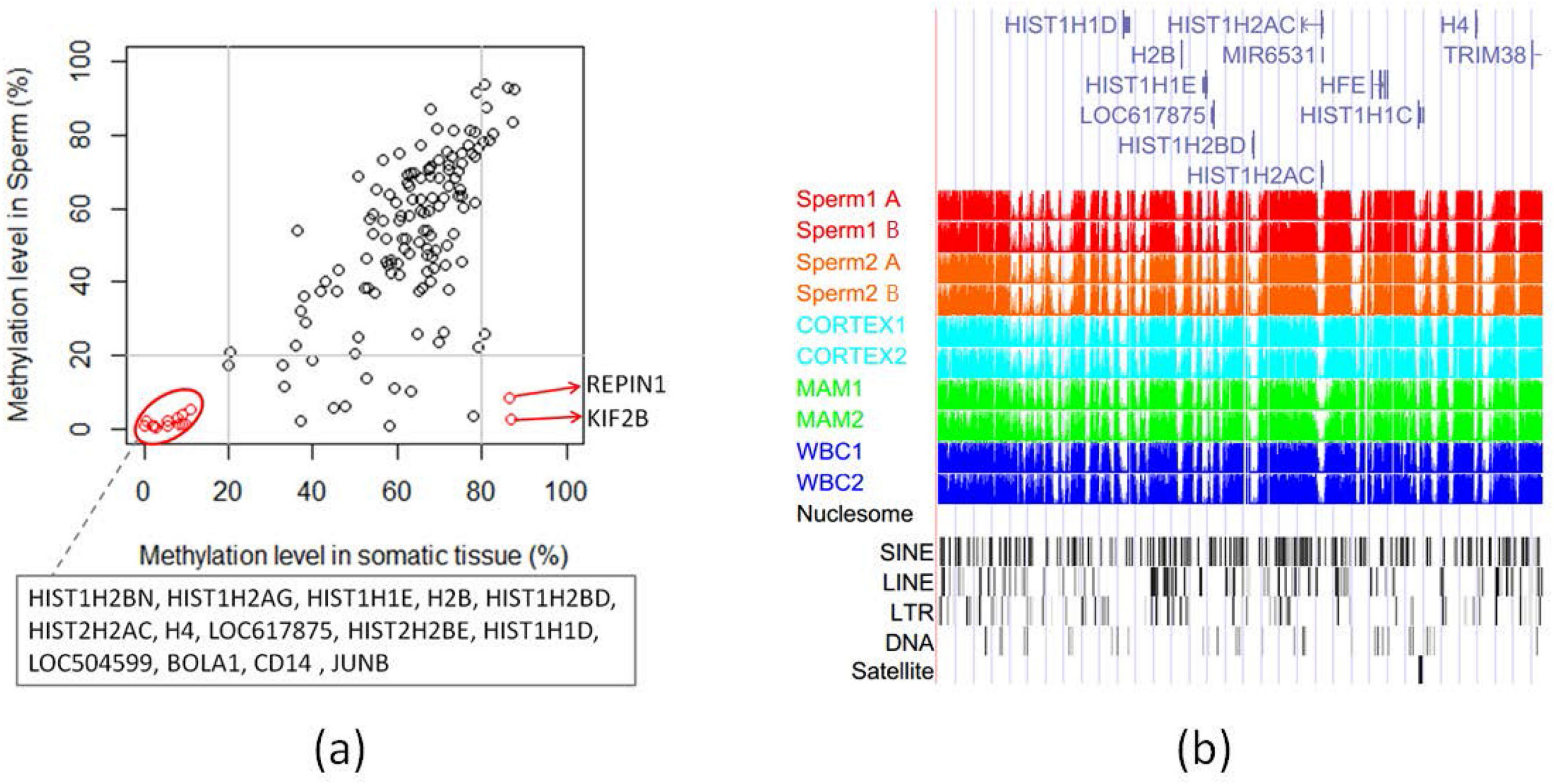
Methylation levels of the genes located within the sperm PMDs. **(a)** Dot plot of the methylation levels of the genes located in the sperm PMDs. Only gene methylation level with standard deviations less than 20% among somatic tissues or sperm cells were used for plotting. **(b)** CpG methylation status of the partial PMD (chr23:30,700,001–31,700,000) clustered genes related to histones. Sperm1 A and B: sperm samples from Holstein 1; Sperm2 A and B: sperm samples from Holstein 2; WBC: whole blood cells; MAM: mammary glands; CORTEX: prefrontal cortex of the brain.

There were 168 genes from the refGene database located in the sperm PMD regions. Gene Ontology (GO) analysis showed that they were significantly enriched in the nucleosome and histone-related GO terms such as chromosome, DNA binding, nucleus, nucleosome core (Supplementary Table S3). The genic methylation level in the PMDs of sperm cells was significantly lower (*P* < 0.01, Student *t* test) than those of somatic tissues (Supplementary Fig. S9). However, the genes seemed to cluster in a few PMDs that appeared in both somatic and sperm cells (Supplementary Table S2). The methylation levels of 11/14 genes related to the histone were commonly hypomethylated (methylation level <20%) in both somatic and sperm cells (Fig. 3a). The histone-related hypomethylated genes, including *HIST1H2AG*, *HIST1H2BN*, *HIST1H1D*, *H2B*, *HIST1H1E*, *HIST1H2BD*, *HIST1H2AC*, *H4*, and *LOC617875*, were clustered in one PMD (chr23:30,700,001–31,700,000, 1 Mb) that were, interestingly, also localized in HMRs (Fig. 3b). When comparing across sperm cells and somatic tissues, we obtained 28 genes that were significantly less methylated (methylation difference >20% and false discovery rate [FDR] <0.01) in sperm cells (Supplementary Table S4). Two of them (*KIF2B* and *REPIN1*), which are involved in meiosis, were found to be hypomethylated in sperm cells but hypermethylated (methylation level >80%) in somatic tissues (Fig. 3a). *KIF2B* has microtubule depolymerization activity and plays a role in chromosome congression. *REPIN1* is required for initiation of chromosomal DNA replication.

### Hypomethylated regions in sperm cells and somatic cells

**Figure 4:**
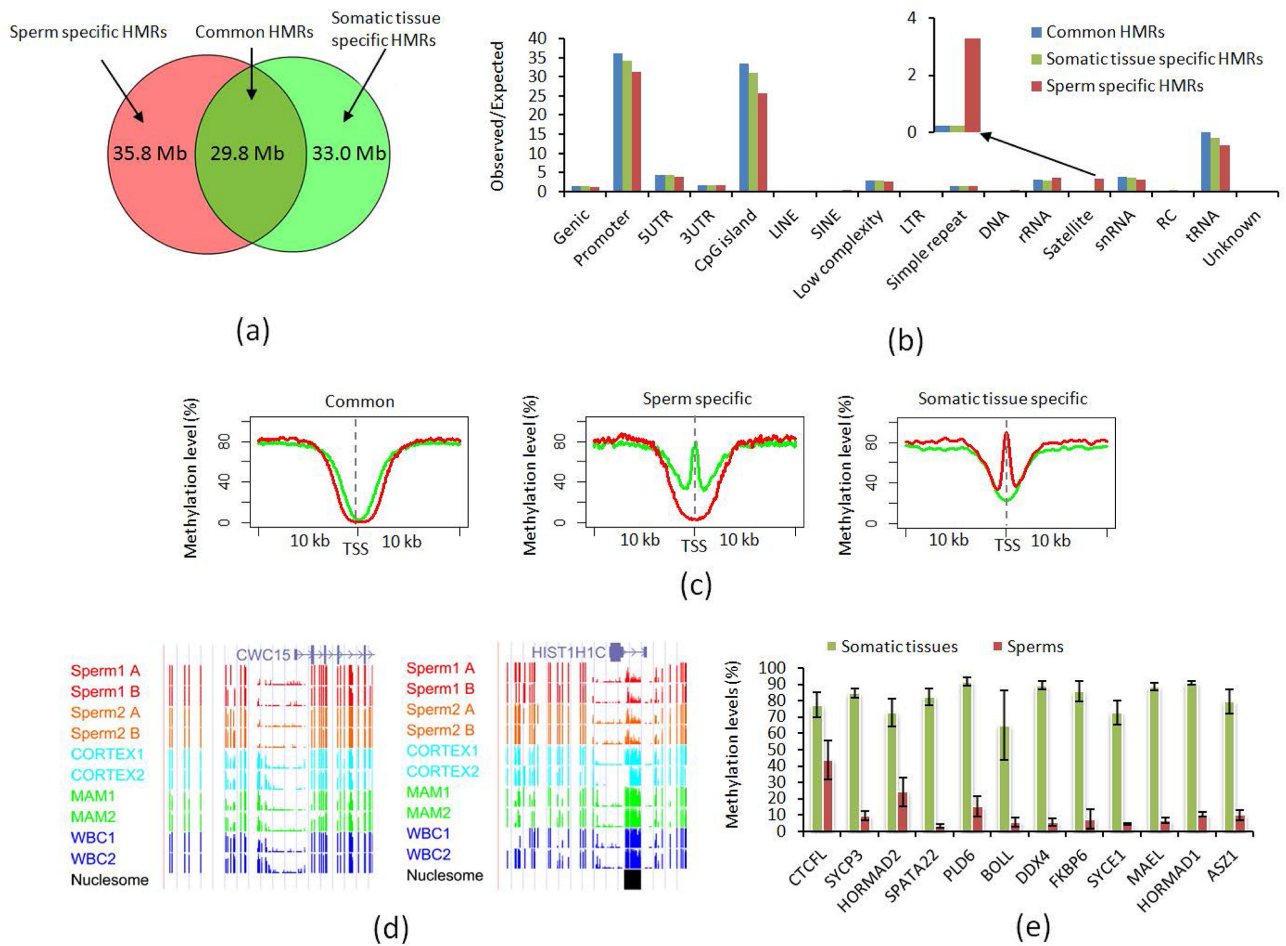
Comparison of HMRs between sperm and somatic cells. **(a)**Venn plot for the HMRs between sperm and somatic cells. **(b)** Genomic feature enrichment analysis in the HMRs that are shared or unique for sperm and somatic cells. **(c)** UP: methylation distribution around the transcription start site (TSS) in the shared HMRs between sperm and somatic cells. DOWN: CpG methylation status of two genes for the nested HMRs around TSS. **(d)** Methylation distribution around the TSS in the sperm- or somatic cell-specific HMRs. **(e)** Methylation levels of the genes with TSS located in the sperm-specific HMRs in sperm and somatic cells. Sperm1 A and B: sperm samples from Holstein 1; Sperm2 A and B: sperm samples from Holstein 2; WBC: whole blood cells; MAM: mammary glands; CORTEX: prefrontal cortex of the brain.

To identify HMRs for sperm and somatic cells, we used a sliding window approach with a window size of 200 bp and extended the window in 50-bp increments until it contained less than 80% hypomethylated (methylation level <20%) CpGs. Using this strict threshold, we observed ∼64 k (65.6 Mb in length) HMRs in sperm and ∼63 k (62.8 Mb in length) HMRs in somatic tissues. In addition to the shared 29.8 Mb of HMRs, nearly half of them (∼35 Mb in sperm cells and ∼33 Mb in somatic cells) were unique to either sperm or somatic cells (Fig. 4a). In sperm, all PMDs were supported by HMRs (with overlap counts ranging from 14 to 567), while only 13.5% HMRs were supported by the PMDs. These findings suggested that there were still large portions of HMRs in either sperm or somatic tissues that were not supported by PMDs, in addition to those regions enriched in PMDs.

Based on the O/E values, the promoter, CGI, and tRNA regions were most enriched in the HMRs (Fig. 4b). Approximately 67% of refGenes had transcription start site (TSS) localized in the HMRs of either sperm or somatic cells. Moreover, more than half of the CGI were overlapped with the promoter regions. This agreed with the long recognized observation that the CGI and regions around TSS are generally hypomethylated. However, similar to PMDs as described above, the positive correlation between the methylation differences (between sperm and somatic cells) and the satellite enrichment in sperm cells was still evident, with a 3.29 O/E value in HMRs (Fig. 4b). On the other hand, the O/E value of the satellite regions, which overlap with somatic tissue-specific HMRs, was only 0.23. We also found that 52.4% of the satellite regions were located in the sperm-specific HMRs, while less than 1% of the satellite regions were located in the somatic tissue-specific HMRs.

We found significant (*P* = 1.97 × 10^−10^, Student's t test) enrichment of the sperm nucleosomes in sperm HMRs than in somatic tissue HMRs (Supplementary Table S10). Of the 5,369 nucleosome peaks in the autosomes of the cattle sperm, 35.4–40.1% were overlapped with sperm HMRs while only 1.5–3.5% were overlapped with somatic tissue HMRs. Moreover, 71.9% of the nucleosome peaks were overlapped with the shared HMRs among different sperm (Supplementary Fig. S10). The sperm nucleosome peaks that overlapped with sperm HMRs were mostly (82.5% in length) composed of satellite sequences with high CG density and low gene or promoter content.

### Distinct characteristics for the shared HMRs in sperm cells and somatic cells

Most of the TSS were commonly hypomethylated in both sperm and somatic cells. We plotted the average methylation around TSS associated with the common HMRs. Similar to the observation for the HMRs in embryonic stem cells compared to sperm in the human study [14], we also observed nested HMRs around TSS in somatic tissues when compared to sperm for the common HMRs (Fig. 4c). This also was supported by the size distributions of HMRs in sperm cells and somatic cells. The mean size of HMRs was ∼729 bp and the median was ∼600 bp in sperm cells. In somatic tissues, the mean size of HMRs was ∼550 bp and the median was ∼450 bp. We then focused on 431 genes that were detected with TSS located in the HMRs of all samples. More than 85.4% of TSS were located in the HMRs of somatic tissues that were nested in at least one side of the sperm HMRs. For example, the extended methylation around the TSS of the *CWC15* gene may affect its regulation of pre-mRNA splicing (Fig. 4d left panel).

### Sperm-specific HMRs were enriched in promoters of genes that were functional in testis

The TSS of 978 genes and 1,275 genes were specifically overlapped with somatic tissue HMRs and sperm HMRs, respectively. Distinct methylation patterns were seen around TSS between sperm cells and somatic tissues (Fig. 4c). The genes with TSS overlapping with somatic cell-specific HMRs were significantly enriched in the functional categories related to immunity, including glycoprotein, immunity, innate immunity, and inflammatory response (Supplementary Table S5). Functional analysis of the genes with TSS specifically overlapping with sperm HMRs illustrated that the genes were related to functions in testis. The most significantly enriched functional category was meiosis (FDR corrected *P* value = 3.3E-4) (Supplementary Table S6). Functional annotation clustering analysis also received the highest enrichment score (1.67) for the GO terms related to functions in testis, including DNA methylation involved in gamete generation, piRNA metabolic process, gene silencing by RNA, and male meiosis (Supplementary Fig. S11).

Further validation confirmed the CG methylation status around the TSS for 12 of the 16 genes involved in testis functions, including *BOLL*, *MAEL*, *ASZ1*, *SYCP3*, *CTCFL*, *MND1*, *SPATA22*, *PLD6*, *DDX4*, *RBBP8*, *FKBP6*, and *SYCE1*. The other four genes were false positives caused by the low density of the CG covered around the TSS. Except for *CTCFL*, the other 11 genes were detected with co-expression using the program STRING according to previous cattle and mouse studies (Supplementary Fig. S12). We precisely defined the boundaries of the sperm-specific HMRs overlapping with TSS of the 12 genes (Supplementary Table S7). Their average methylation levels were significantly lower in sperm cells than the somatic cells (Fig. 4e). Moreover, these low methylated regions were strongly enriched for putative binding sites of transcription factors such as E2F1, E2F6, and NRF1, which are known to function in testis (Supplementary Fig. S13). We found all 12 genes had CGI-associated hypomethylation. However, the low methylation was not restricted to the CGI region but extended to a much larger region including repeat elements (Supplementary Table S7).

We also checked to see if the nucleosome peaks overlap with the sperm-specific HMRs in the promoter regions. We did not observe overlaps for the above 12 genes involved in the testis functions but found overlaps for 5 other genes (*TUFT1, WRN, RAB11FIP5, RPS6*, and *HIST1H1C*) related to the GO terms of protein modification and localization. For example, *HIST1H1C* is involved in acetylation, methylation, and phosphoprotein. A nucleosome peak was found in the first intron, where there is low methylation in sperm and high methylation in somatic tissues (Fig. 4d left panel).

### Hypomethylated BOV-A2 were enriched around the TSS in sperm cells

Most of the repeat elements, especially retrotransposons, showed high methylation levels that are required for transcriptional silencing. Similar to studies in other species, the elements that remain active in cattle, such as long interspersed nuclear element (LINE)/RTE-BovB and LINE/L1, displayed high methylation levels even at high CG density (≥5%) in both sperm and somatic cells. Moreover, we found that methylation levels in BovB elements negatively correlated with their sequence divergence from their consensus sequence, thus their evolutionary age (Supplementary Fig. S14).

**Figure 5:**
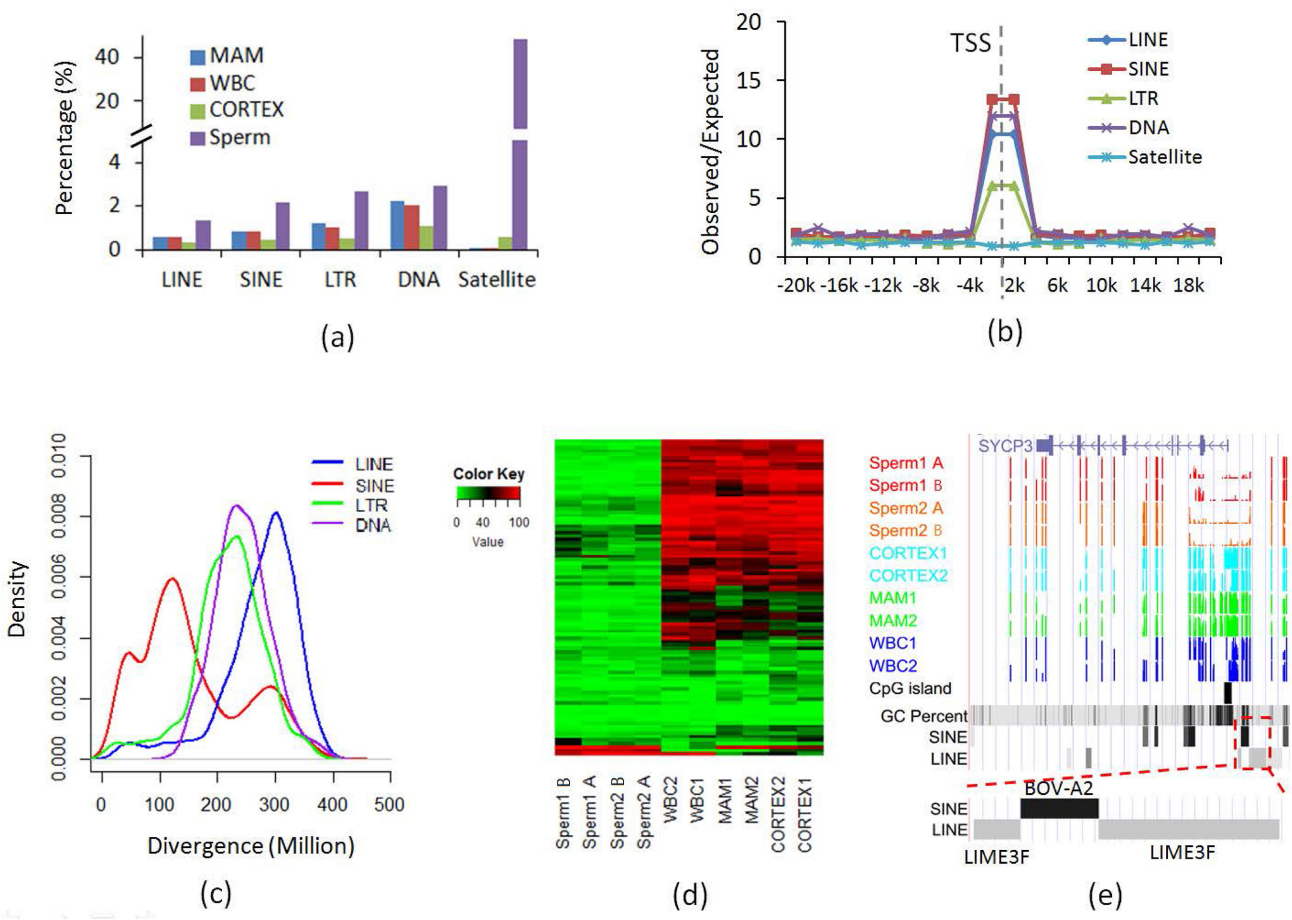
Analysis of the hypomethylated repeats. **(a)** Percentage of hypomethylated elements for common repeats. **(b)** Enrichment of the hypomethylated repeats around TSS. **(c)** Sequence divergence and thus age distribution of common repeats (*x*-axis: % substitutions in matching region compared to the consensus). *(d)* Heat map plot for the methylation levels of hypomethylated BOV-A2 in sperm and somatic cells. Each row represents one BOV-A2 element. *(e)* An example of the BOV-A2 element inserted in the region around TSS near the *SYCP3* gene. Sperm1 A and B: sperm samples from Holstein 1; Sperm2 A and B: sperm samples from Holstein 2; WBC: whole blood cells; MAM: mammary glands; CORTEX: prefrontal cortex of the brain.

However, there were still some repeats that were hypomethylated (Fig. 5a). We extracted the elements that were at least hypomethylated in one sample for LINE, SINE, LTR, DNA, and satellite. The hypomethylated repeats (LINE, SINE, LTR, and DNA) other than satellite were highly enriched within 2 kb of the TSS (Supplementary Fig. 5b). The hypomethylated elements had higher CG density and overlapped or were near at least one CGI (Supplementary Fig. S15a). The hypomethylated elements had higher levels of DNA methylation variation, which implied their potential function in gene expression regulation (Supplementary Fig. S15b). When we checked the age of the hypomethylated elements, we found that only the hypomethylated elements in SINE regions were still associated with young age, while the elements in LINE, LTR, and DNA repeat classes were associated with old age (Fig. 5c).

We selected the hypomethylated elements with sequence divergence less than 50 in SINE and found 96.8% (675/697) were BOV-A2. The lengths of the young BOV-A2 were enriched between 90 and 100% of its consensus sequence, supporting the young age of those hypomethylated BOV-A2. The young BOV-A2 with low methylation may be active, especially near the TSS region, which may change the promoter structure by introducing new transcription factor binding sites (TFBSs). We found 31 genes with hypomethylated BOV-A2 located within 2 kb of their TSS (Supplementary Table S8). Most of the candidate BOV-A2 showed specific hypomethylation in sperm cells, which illustrated that they may be active in certain developmental stages (Fig. 5d). For example, *SYCP3*, one gene functional in spermatogenesis, was found to have a BOV-A2 inserted into an ancient LIME3F element separating it into two parts (Fig. 5e). Further searching for TFBSs in the BOV-A2 sequence found multiple TFBSs, and some of them were with function in the testis (Supplementary Table S9).

## Discussion

Using WGBS, we generated one of the first single-nucleotide resolution cattle sperm DNA methylomes and compared them to the cattle somatic tissue methylomes. The global CG methylation levels detected ranged from 72.8 to 78.1% among our cattle samples, which were similar to those in other mammalian species such as humans (∼70%)but significantly higher than the earlier reduced representation bisulfite sequencing (RRBS) results (approximately 30–40%) [14, 47]. It is important to point out that RRBS only reports on the CG-enriched regions of the genome. The most comprehensive methods such as WGBS provide a more representative global estimate. Our genome-wide cattle methylomes confirmed existing knowledge that DNA methylation is important for gene expression and plays a critical role in tissue-specific processes [5, 51]. In promoter regions, DNA methylation is associated with transcriptional repression, whereas in gene bodies, DNA methylation is generally enriched in the body of highly transcribed genes [52–56]. As reported for other mammals, global resetting of DNA methylation patterns happens twice during development: once during germ cell development and once during early embryogenesis. Our data permit a genome-wide analysis of the first reprogramming event in cattle.

### Partially methylated domains

PMDs are large domains of DNA (often greater than 100 kb) that have lower levels of DNA methylation. They were first discovered and defined in cultured human fibroblasts [57]. PMDs were later described in human cancer cells, most mammalian placenta, and mouse germline cells [58–62]. They are often associated with inaccessible chromatin and inactive histone marks, covering entire genes and gene clusters. The mechanisms of PMD formation and the biological significance of PMDs have yet to be determined; one possibility is that they mark the locations for repressing tissue-specific genes in the inappropriate cell type.

In this study, we found that cattle sperm PMDs share features with those identified in other cell types, especially those identified in mouse germline cells: localization in genomic regions with low GC contents, low CGI density, and low gene density. Thus, we speculated that a similar silencing mechanism may operate in cattle sperm PMDs because they share genomic localizations and structural features with the other PMDs. The existence of PMDs in cattle sperm cells, but rarely in the somatic tissues, is consistent with our observation that sperm DNA tends to have more hypomethylated CG sites in low GC content regions than in the somatic tissues [62]. In our cattle sperm cells, genes in PMDs commonly included lowly methylated gene clusters related to histone. We also found that genes hypomethylated in sperm but hypermethylated in somatic tissue had testis- or sperm-specific functions. For example, *KIF2B* has microtubule depolymerization activity and plays a role in chromosome congression. Therefore, HMRs could be greatly involved in the biological process of gene expression.

### Hypomethylated regions

We detected large differences between sperm cells and somatic tissues in terms of HMRs. HMRs often occur in CGIs; however, they also occur outside of CGIs and function as cell type-specific enhancers. As has been reported [63–65], the formation of HMRs can be due to two possible mechanisms: active transcription and accompanying histone marks such as H3K4me3 prevent the access of DNA methyltransferases and specific protein/DNA complexes, such as CTCF and Sp1, inhibit the methylation machinery in the absence of transcription.

The retained nucleosomes and their post-translational modifications represent potential mediators of epigenetic information transmitted from the sire to its offspring via sperm. In our bovine dataset, the sperm nucleosome peaks that overlapped with sperm HMRs were mostly composed of satellite sequences with high CG density and low gene or promoter content, providing evidence for the predominant retention of sperm nucleosomes in gene deserts.

For shared HMRs, we also observed the “nested” HMR phenomenon as described previously [14], in which the HMRs in sperm cells were larger than those in somatic cells. The function of those genes in our study was significantly enriched in the terms related to epigenetics, such as acetylation, phosphoprotein, and mRNA splicing.

For genes overlapped by sperm-specific HMRs, we found the enrichment of GO terms related to male germ cell processes, including DNA methylation involved in gamete generation, piRNA metabolic process, gene silencing by RNA, and male meiosis. We further identified the 12 genes whose TSS overlapped with sperm-specific HMRs (Supplementary Table S7). For example, the *BOLL* gene belongs to the DAZ gene family required for germ cell development. Loss of this gene function results in azoospermia and male infertility [66]. Acting via the piRNA pathway, genes *ASZ1*, *MAEL*, and *PLD6* play a central role during spermatogenesis by repressing transposable elements and preventing their mobilization, which is essential for germline integrity [66–70]. The *CTCFL* gene is a paralog of *CTCF* and appears to be expressed primarily in the cytoplasm of spermatocytes, whereas *CTCF* is expressed primarily in the nucleus of somatic cells [71]. Although CTCF forms methylation-sensitive insulators that regulate chrX inactivation, the CTFCL protein correlates with resetting of methylation marks during male germ cell differentiation. Genes such as *SYCE1* and *SYCP3* encode structural components of the synaptonemal complex, which is involved in synapsis, recombination, and segregation of meiotic chromosomes [72, 73]. *MND1* and *SPATA22* encode proteins required for homologous recombination in meiosis [74, 75]. *DDX4*, a DEAD box protein, characterized by the conserved motif Asp-Glu-Ala-Asp (DEAD), encodes a putative RNA helicase, which is specifically expressed in the germ cell lineage in both sexes and functions in germ cell development [76].

### Common repeats

In germ cells such as sperm, common repeats are normally highly methylated. The conserved piRNA pathway has been proposed to be important for recognizing and silencing repeats in germ cells [77]. However, we still found more than expected HMRs that overlapped common repeats in sperm cells, suggesting some individual elements can evade piRNA-based silencing. Examining patterns of HMR-associated repeats is very informative. One possibility is that as with genes, young repeats contain promoters or regulatory regions and/or their TF binding and transcription activation can facilitate their evading default methylation. Although most BOV-A2 elements follow the neutral expectation, showing a negative correlation between methylation level and age (represented by their divergence from its consensus sequence), we detected that some BOV-A2 elements were hypomethylated in cattle sperm cells. Similar to the young Alu subfamilies that introduce binding sites for transcription factor SABP in human sperm [78, 79], we found some BOV-A2 elements inserted into genes such as *SYCP3*, which itself is involved in spermatogenesis. By examining these Bov-A2 insertions, we found the binding sites for multiple TFs that have functions in testis. As the introduction of TFBS by active Bov-A2 insertions could change the promoter structure, we hypothesize that Bov-A2 insertions in sperm cells may be involved in specific regulation of functional genes. Our results also were consistent with earlier studies, supporting the existence of a system based on environmental and epigenetic signals that is able to spread and mutate the Bov-A2 sequence in the genes expressed during the response to cellular activation signals [80]. Through this adaptive mechanism, ruminants may reinforce and diversify the stress reaction at cellular and individual levels in response to environmental changes.

### Centromeric satellites

In cattle sperm, we found heavy selective hypomethylation of megabase domains of centromeric satellite clusters, as compared to satellites located elsewhere, which were generally methylated at medium levels. Our results also supported the following proposition, in which these regions were initially hypomethylated in male germline cells and then shifted to the hypermethylation status during differentiation into somatic lineage. This agreed with previous observations made in human and mouse [14, 81], confirming a conserved epigenetic signature for which the chromosomal centromeric and pericentric regions in male germline cells are specifically hypomethylated, despite the hypermethylation status in somatic cells. These observations were consistent with the hypothesis that maintaining hypomethylation of satellites in centromeres might be critical for chromosome segregation during meiosis and their rapid transcriptional activation upon fertilization [82].

It is noted that we used at least 10 × coverage to call methylation differences at single CG site level. Only when we did the analysis at the region level (PMD, HMR, or elements), we used the CG sites with more than 5 × coverage to calculate the methylation level. As reported previously, Ziller et al. used several high-coverage reference datasets to experimentally determine minimal sequencing requirements to be between 5 and 15 × coverage per sample [83]. They further discussed the trade-off between sequencing depth and number of assayed replicates. In this study, we chose to sequence each of two biological replicates to 18 ×, instead of sequencing one sample to 30 ×. Additionally, 5 × or even lower cutoffs had been used before for methylation analysis at the region level [83–86]. Since all 5 × CG sites detected in one region across multiple samples were considered simultaneously, the statistical power was enhanced, even in the presence of stochastic variation of high-throughput sequencing.

In summary, we provided baseline methylation profiles for cattle sperm and somatic cells at a single-base resolution. We characterized the DNA methylome and assessed DNA methylation patterns. We reported rich datasets of PMDs and HMRs across different tissues and detected a subset of them that correlated with tissue development. Our study contributes to the understanding of cattle DNA methylation patterns and provides foundational information for further investigations.

## Methods

### Sample collection and DNA isolation

Somatic tissues including parenchymal tissue from the mammary glands, whole blood cells, and prefrontal cortex of the brain were collected from two healthy adult Holstein cows (3 to 4 yearsold; one lactating and one nonlactating), snap frozen in liquid N_2_ immediately after excision, and kept at –80°C until use. Semen straws were collected twice from two fertile Holstein bulls. Genomic DNA for each tissue was isolated according to the QIAamp DNA Mini Kit protocol (QIAGEN, Valencia, CA, USA). The quality of DNA samples was evaluated using the 2100 Bioanalyzer (Agilent Technologies, Santa Clara, CA, USA) including degradation, potential RNA contamination, purity (OD260/OD280), and concentration using a spectrophotometer (NanoDrop Technologies, Rockland, DE) to meet the requirements for library construction.

### Library construction and sequencing

The qualified genomic DNA from somatic tissues and sperm were used to construct libraries. Briefly, 3 μg of genomic DNA spiked with unmethylated lambda DNA were fragmented into 200–300 bp using a Covaris S220 (Covaris, Inc., Woburn, MA, USA), followed by terminal repairing and A-ligation. Different cytosine methylated barcodes were ligated to sonicated DNA for different samples. The DNA bisulfite conversion was performed using the EZ DNA Methylation Gold Kit (Zymo Research, Irvine, CA, USA). Then single-stranded DNA fragments were amplified using the KAPA HiFi HotStart Uracil + ReadyMix (2 X) (Kapa Biosystems, Wilmington, MA, USA). The library concentration was quantified using a Qubit 2.0 fluorometer (Life Technologies, Carlsbad, CA, USA) and qPCR (iCycler, BioRad Laboratories, Hercules, CA, USA), and the insert size was checked using the Agilent 2100. To decrease the batch effect, the libraries for one sample were balanced, mixed with other libraries with different barcodes, and sequenced on different lanes of a HiSeq X Ten (Illumina, San Diego, CA, USA) to generate 150-bp paired-end reads by Novogene (Novogene, Beijing, China).

### Sequence alignment and identification of methylcytosine

Programs FastQC v 0.11.2 (FastQC, RRID:SCR_014583) and Trim Galore v 0.4.0 (Trim Galore, RRID:SCR_011847) were used to generate sequence quality reports and to trim/filter the sequences, respectively [87]. For each sample, high-quality reads were obtained after trimming low-quality bases and the adapter sequences. The cleaned data for each sample were merged and aligned to the reference genome (*Bos taurus* UMD3.1 [88]) using bowtie2 under the Bismark software (0.14.5) with the parameters -p 3 -N 1 -D 20. The methylcytosine information was extracted using the bismark_methylation_extractor after deduplicating the duplication reads. The first 6 bp were ignored for the paired-end reads to decrease the potential effects of severe bias toward nonmethylation in the end-of-reads caused by end repairing.

### Global comparison between methylomes of sperm cells and somatic cells

The common CGs with depth greater than 10 × among all sample were used for global comparison between methylomes of sperm cells and somatic cells. Cluster analysis, PCA analysis, and DMC detection were applied using a R package (methykit, R version 3.3.3) [89]. The DMCs were defined as the methylation difference greater than 30% and *q* value <0.01 between sperm cells and somatic cells. The genome structure annotation files for refGene, CGI, repeats were downloaded from the UCSC database [90]. The promoter regions were defined as ±1000 bp around the transcript start sites. The methylation levels for each element in different genomic features were calculated as the average methylation level of the CGs with at least 5 × coverage. Only the elements that met the following criteria were used for further analysis: at least 10% CG detection rate for elements with more than 50 CGs and at least five CGs detected for elements with fewer than 50 CGs. R packages were used to plot the comparison results.

### PMD and HMR identification

All the CGs with at least 5 × coverage were used for PMD and HMR detection. For PMD detection, we first calculated the average methylation level for each of the nonoverlapped 20-kb windows. According to the distribution of the windows’ average methylation (Supplementary Fig. S5a), we selected 60% methylation as the threshold to divide the windows after several trials using methylation levels at 50%, 55%, 60%, 65%,and 70%. The windows with methylation level greater than 60% were assigned a 1 and the windows with methylation level less than 60% were assigned a 0. Then, we applied a hidden Markov model using HMM (one R package at [91]) to detect the windows assigned with continuous 0 for each sperm sample. The sperm PMDs used in this study had to meet the following criteria: supported by at least three sperm samples and combined from at least three windows.

To identify the contiguous HMRs for sperm cells and somatic cells, we used a sliding window approach with a window size of 200 bp and extended the window by 50-bp steps until it contained less than 80% hypomethylated (methylation level <20%) CpGs. Only the HMRs with at least 5 CG detected with more than 5 × coverage were used for analysis. The GenomicRanges package in R was used to calculate statistics of the overlapped HMRs in different tissues or sperm.

### Sperm nucleosome location detection

Chip-seq data for sperm nucleosome-binding sites detection was downloaded from the Gene Expression Omnibus database with the accession number GSM1160360. The sample preparation procedure can be found in [92]. NGSQCToolkit (version 2.3.3) software was used to filter the adapters and low-quality reads. Then, the qualified reads were aligned to the reference genome (*Bos taurus* UMD3.1) using bowtie2 (version 2.3.3; -N 0 -L 22 -i S,1,1.15 –dpad 15 -gbar 4),and peaks were called using MACS (version 1.4.2; –keep-dup 1 –wig –single-profile –space = 10 –diag) with default parameters. We detected 5,369 nucleosome location peaks in the autosome of cattle sperm (Supplementary Table S10).

### Gene function analysis

Gene functional annotation analyses were applied using the online DAVID software [93]. Fisher exact test was used to measure gene enrichment in annotation terms. *P* values were corrected by FDR to search for significantly enriched terms. The STRING software [94] with default parameters was used to extract co-expressed genes using cattle and mouse databases because of fewer supporting data available in the cattle database. We used Homer software [95] to detect enriched motifs within 1,000 bp up- and downstream of the TSS for genes involved in spermatogenesis. At the same time, the sequences within 1,000 bp up- anddown stream of the TSS for refGene were used as background. We used a website software (Genomatrix, [96]) to search for TFBS in the Bov-A2 sequence.

## Availability of supporting data

WGBS data are available from GEO under the accession number GSE106538. Supporting data are also available via the *GigaScience* repository, GigaDB [97].

## Additional files

Additional file 1.

Figure S1: Cluster analysis according to the CpG methylation.

Figure S2: Principal component analysis based on CpG methylation.

Figure S3: Global comparisons of distinct genomic features between sperm cells and somatic cells in cattle.

Figure S4: Methylation level distribution histograms and heat map plots for selected genomic features.

Figure S5: Methylation level distribution histograms for sperm cells and somatic cells.

Figure S6: Distribution of partially methylated domains (PMDs) within the cattle genome.

Figure S7: Enrichment of different genomic features by calculating the Observed/Expected ratio between the observed density in sperm-specific PMDs and the average density in autosomes.

Figure S8: Heat map plot for the methylation levels of satellite-containing PMDs in sperm and somatic cells.

Figure S9: The genic methylation level in the PMD of sperm cells was significantly lower (*P* < 0.01, student's t test) than those of somatic cells.

Figure S10: Venn plot for overlaps of HMR-associated nucleosomes among four sperm samples.

Figure S11: Functional annotation clustering analysis.

Figure S12: Functional analysis for the genes with TSS specifically overlapped with sperm HMRs illustrated that the genes were related to functions in testis.

Figure S13: Sperm-specific HMRs were enriched for transcription factor binding sites known to function in the testis.

Figure S14: Methylation levels in BovB elements negatively correlated with their divergence thus their evolutionary age.

Figure S15: The hypomethylated elements had higher CG density and overlapped or were near at least one CpG island (a). The hypomethylated elements were with higher levels of DNA methylation variation which implies their potential function in gene expression regulation (b).

Additional file 2.

Table S1: Differentially methylated cytosines between sperm and somatic tissues.

Table S2: Information of partial methylated domains in sperm.

Table S3: Significantly enriched GO terms for the genes located in the PMDs.

Table S4: Differentially methylated genes in PMDs between sperm and somatic tissues.

Table S5: Significantly enriched GO terms for the genes located in the somatic tissue specific HMRs.

Table S6: Significantly enriched GO terms for the genes located in the sperm specific HMRs.

Table S7: Information of sperm specific HMRs overlapped with TSS of the 12 genes functioning in testis.

Table S8: Genes overlapped with hypomethylated young BOV-A2 in the promoter region.

Table S9: Transcription factor binding sites in the BOV-A2 sequence.

Table S10: Nucleosome location peaks in the autosome of cattle sperm.

## Abbreviations

CGI, CpG island; DMC, differentially methylated cytosine; FDR, false discovery rate; GO, Gene Ontology; HMR, hypomethylated regions; LINE, long interspersed nuclear element; LTR, long terminal repeat; O/E, observed/expected; PCA: principle component analysis; PMD, partially methylated domain; RRBS, reduced representation bisulfite sequencing; SINE, short interspersed nuclear element; TFBS, transcription factor binding site; TSS, transcription start site; WGBS, whole genome bisulfite sequencing.

## Ethics approval

All samples were collected with approval of the US Department of Agriculture Agriculture Research Service Institutional Animal Care and Use Committee under Protocol 16-016.

## Competing interests

The authors declare that they have no competing interests.

## Supplementary Material

GIGA-D-17-00303_Original_Submission.pdf

GIGA-D-17-00303_Revision_1.pdf

GIGA-D-17-00303_Revision_2.pdf

Response_to_Reviewer_Comments_Original_Submission.pdf

Response_to_Reviewer_Comments_Revision_1.pdf

Reviewer_1_Report_(Original_Submission) -- Ole Madsen 12/11/2017 Reviewed

Reviewer_1_Report_(Revision_1) -- Ole Madsen 2/24/2018 Reviewed

Reviewer_2_Report_(Original_Submission) -- Stephanie McKay 1/23/2018 Reviewed

Supplemental material
