# Supplementary material for: Comparative whole genome DNA methylation profiling of cattle sperm and somatic tissues reveals striking hypomethylated patterns in sperm
Source: Gigascience. 2018 Apr 10;7(5):giy039. doi: 10.1093/gigascience/giy039 (PMC5928411; doi:10.1093/gigascience/giy039)
Supplement: GIGA-D-17-00303_Original_Submission.pdf [file giy039_giga-d-17-00303_original_submission.pdf]

GigaScience

Genome-wide sequencing and comparative profiling of cattle sperm DNA methylome reveals its hypomethylated patterns

--Manuscript Draft--

**Manuscript Number:**	GIGA-D-17-00303
**Full Title:**	Genome-wide sequencing and comparative profiling of cattle sperm DNA methylome reveals its hypomethylated patterns
**Article Type:**	Research
**Funding Information:**	National Institute of Food and Agriculture (2013-67015-20951) — Dr. George Liu<br>US-Israel Binational Agricultural Research and Development (BARD) Fund (US-4997-17) — Dr. George Liu
**Abstract:**	Compared to somatic cells, sperm cells undergo nearly complete reprogramming of DNA methylation and exchange histones by protamine. Although sperm DNA methylation has been studied in humans and other model species, as the most beneficiary species of artificial insemination, its status in cattle is largely unknown. Using whole-genome bisulfite sequencing (WGBS), we profiled the DNA methylome of cattle sperm through comparison with somatic cells from three bovine tissues (mammary gland, brain, and blood). Large differences between cattle sperm and somatic cells were observed in the methylation patterns of global CpGs, pericentromeric satellites, partially methylated domains (PMDs), hypomethylated regions (HMRs), and common repeats. As expected and like other species, we observed low methylation in the promoter regions and high methylation in the bodies of active genes. We detected selective hypomethylation of megabase domains of centromeric satellite clusters, which may be related to chromosome segregation during meiosis and their rapid transcriptional activation upon fertilization. We found more PMDs in sperm cells than in the somatic cells and identified meiosis-related genes like KIF2B and REPIN1, which are hypomethylated in sperm but hypermethylated in somatic cells. Besides the common HMRs around gene promoters which showed substantial differences between sperm and somatic cells, the sperm-specific HMRs also targeted to distinct spermatogenesis-related genes, including BOLL, MAEL, ASZ1, SYCP3, CTCFL, MND1, SPATA22, PLD6, DDX4, RBBP8, FKBP6, and SYCE1. Although common repeats were heavily methylated in both sperm and somatic cells, some hypomethylated repeats were enriched in gene promoters with large variations among tissues. For example, some young Bov-A2 repeats, which belong to the SINE family, were hypomethylated and could affect the promoter structures by introducing new regulatory elements. Our study provides a comprehensive resource for bovine sperm epigenomic research and enables new discoveries about DNA methylation and its role in male fertility.
**Corresponding Author:**	George Liu<br><br>UNITED STATES
**Corresponding Author Secondary Information:**	
**Corresponding Author's Institution:**	
**Corresponding Author's Secondary Institution:**	
**First Author:**	Yang Zhou
**First Author Secondary Information:**	
**Order of Authors:**	Yang Zhou
	Erin Connor
	Derek Bickhart

	Congjun Li
	Ransom Baldwin
	Steven Schroeder
	Benjamin Rosen
	Liguo Yang
	Curtis Van Tassell
	George Liu
**Order of Authors Secondary Information:**	
**Opposed Reviewers:**	
**Additional Information:**	
**Question**	**Response**
Are you submitting this manuscript to a special series or article collection?	No
**Experimental design and statistics**<br><br>Full details of the experimental design and statistical methods used should be given in the Methods section, as detailed in our Minimum Standards Reporting Checklist. Information essential to interpreting the data presented should be made available in the figure legends.<br><br>Have you included all the information requested in your manuscript?	Yes
**Resources**<br><br>A description of all resources used, including antibodies, cell lines, animals and software tools, with enough information to allow them to be uniquely identified, should be included in the Methods section. Authors are strongly encouraged to cite Research Resource Identifiers (RRIDs) for antibodies, model organisms and tools, where possible.<br><br>Have you included the information requested as detailed in our Minimum Standards Reporting Checklist?	Yes
**Availability of data and materials**<br><br>All datasets and code on which the conclusions of the paper rely must be either included in your submission or deposited in publicly available repositories	Yes

(where available and ethically appropriate), referencing such data using a unique identifier in the references and in the "Availability of Data and Materials" section of your manuscript.<br><br>Have you have met the above requirement as detailed in our Minimum Standards Reporting Checklist?	

Powered by Editorial Manager® and ProduXion Manager® from Aries Systems Corporation

Manuscript Click here to download Manuscript tDNAm_v9.docx

Genome-wide sequencing and comparative profiling of cattle sperm DNA methylome reveals its hypomethylated patterns

Yang Zhou[1,2], Erin E. Connor[2], Derek M. Bickhart[3], Congjun Li[2], Ransom L. Baldwin[2], Steven G. Schroeder[2], Benjamin D. Rosen[2], Liguo Yang[1], Curtis P. Van Tassell[2] and George E. Liu[2,*]

[1]Key Laboratory of Agricultural Animal Genetics, Breeding and Reproduction, Education Ministry of China, Huazhong Agricultural University, Wuhan, Hubei, 430070, China;

[2]Animal Genomics and Improvement Laboratory, BARC, USDA-ARS, Beltsville, Maryland 20705, USA;

[3]The Cell Wall Utilization and Biology Laboratory, USDA-ARS, Madison, Wisconsin, 53706, USA;

Running title: cattle sperm DNA methylome

Yang Zhou, Key Laboratory of Agricultural Animal Genetics, Breeding and Reproduction, Education Ministry of China, Huazhong Agricultural University, Wuhan, Hubei, 430070, China, zhouyang19880528@126.com

Erin E. Connor, Animal Genomics and Improvement Laboratory, USDA-ARS, Beltsville, Maryland 20705, USA, Erin.Connor@ars.usda.gov

Derek M. Bickhart, The Cell Wall Utilization and Biology Laboratory, USDA-ARS, Madison, Wisconsin, 53706, USA, Derek.Bickhart@ars.usda.gov

Congjun Li, Animal Genomics and Improvement Laboratory, USDA-ARS, Beltsville, Maryland 20705, USA, Congjun.Li@ars.usda.gov

Ransom L. Baldwin, Animal Genomics and Improvement Laboratory, USDA-ARS, Beltsville, Maryland 20705, USA, Ransom.Baldwin@ars.usda.gov

Steven G. Schroeder, Animal Genomics and Improvement Laboratory, USDA-ARS, Beltsville, Maryland 20705, USA, Steven.Schroeder@ars.usda.gov

Benjamin D. Rosen, Animal Genomics and Improvement Laboratory, USDA-ARS, Beltsville, Maryland 20705, USA, Ben.Rosen@ars.usda.gov

Liguo Yang, Key Laboratory of Agricultural Animal Genetics, Breeding and Reproduction, Education Ministry of China, Huazhong Agricultural University, Wuhan, Hubei, 430070, China, yangliguo2006@qq.com

Curtis P. Van Tassell, Animal Genomics and Improvement Laboratory, USDA-ARS, Beltsville, Maryland 20705, USA, Curt.Vantassell@ars.usda.gov

George E. Liu Animal Genomics and Improvement Laboratory, USDA-ARS, Beltsville, Maryland 20705, USA, George.Liu@ars.usda.gov

*Corresponding Author:

GEL: Animal Genomics and Improvement Laboratory, USDA-ARS, Building 306, Room 111, BARC-East, Beltsville, MD 20705, USA. E-mail: George.Liu@ars.usda.gov, Voice Phone: +1-301-504-9843, Fax: +1-301-504-8414

Abstract

Compared to somatic cells, sperm cells undergo nearly complete reprogramming of DNA methylation and exchange histones by protamine. Although sperm DNA methylation has been studied in humans and other model species, as the most beneficiary species of artificial insemination, its status in cattle is largely unknown. Using whole-genome bisulfite sequencing (WGBS), we profiled the DNA methylome of cattle sperm through comparison with somatic cells from three bovine tissues (mammary gland, brain, and blood). Large differences between cattle sperm and somatic cells were observed in the methylation patterns of global CpGs, pericentromeric satellites, partially methylated domains (PMDs), hypomethylated regions (HMRs), and common repeats. As expected and like other species, we observed low methylation in the promoter regions and high methylation in the bodies of active genes. We detected selective hypomethylation of megabase domains of centromeric satellite clusters, which may be related to chromosome segregation during meiosis and their rapid transcriptional activation upon fertilization. We found more PMDs in sperm cells than in the somatic cells and identified meiosis-related genes like *KIF2B* and *REPIN1*, which are hypomethylated in sperm but hypermethylated in somatic cells. Besides the common HMRs around gene promoters which showed substantial differences between sperm and somatic cells, the sperm-specific HMRs also targeted to distinct spermatogenesis-related genes, including *BOLL*, *MAEL*, *ASZ1*, *SYCP3*, *CTCFL*, *MND1*, *SPATA22*, *PLD6*, *DDX4*, *RBBP8*, *FKBP6*, and *SYCE1*. Although common repeats were heavily methylated in both sperm and somatic cells, some hypomethylated repeats were enriched in gene promoters with large variations among tissues. For example, some young Bov-A2 repeats, which belong to the SINE family, were hypomethylated and could affect the promoter structures by introducing new regulatory elements. Our study provides a comprehensive resource for bovine sperm epigenomic research and enables new discoveries about DNA methylation and its role in male fertility.

Key words: cattle, sperm, somatic cells, DNA methylation, hypomethylated region, WGBS (Whole genome bisulphite sequencing)

Introduction

DNA methylation plays important roles in normal development and is associated with many processes like gene expression, genomic imprinting, repression of transposable elements, and gametogenesis [1-5]. DNA methylation changes dramatically during mammalian development and aberrant methylation patterns may lead to numerous diseases [6,7]. Compared to somatic cells, sperm cells undergo nearly complete reprogramming of DNA methylation and exchange histones by protamine [8-14]. Sperm DNA methylation patterns have been well characterized in a few species, including humans and rodents [15-20]. These studies found that proper DNA methylation in sperm is required for successful meiosis [21]. In humans, sperm DNA has eight times more hypomethylated loci than DNA from other somatic cells [14,22]. Additionally, sperm DNA hypermethylation has been associated with poor sperm parameters, idiopathic male infertility, and even pregnancy failure [23-29].

Transposable elements or common repeats constitute roughly half of most mammalian genomes [30]. Repression of these common repeats relies on DNA methylation via the piRNA pathway and is essential for the maintenance of genomic stability in the long term and for germ cell function in the short term [31,32]. In humans, common repeats were found to be heavily methylated – with the notable exclusion of young AluY and AluYa5 elements in human sperm cells [33]. If methylation is lost on certain repressed repeats, germ cell development is arrested in meiosis.

Our knowledge of DNA methylation patterns in livestock is still limited when compared to humans and other model species. A few DNA methylation studies were reported with limited tissue types and low resolution in cattle, pigs, sheep, and horses [34-48]. As the species benefitting most from artificial insemination, we aimed to profile the cattle sperm DNA methylome through comparison with somatic cells from three tissues (mammary gland, brain, and blood). We constructed their DNA methylation profiles using the whole genome bisulfite sequencing (WGBS) method. We investigated the landscapes of the DNA methylome in sperm as compared to the somatic cells. We studied differential methylation by comparing them in multiple contexts, including global CpGs, pericentromeric satellites, partially methylated domains (PMDs), hypomethylated regions (HMRs), and common repeats. In line with the Functional Annotation of Animal Genome (FAANG) project [49], this study provides a comprehensive resource for bovine sperm epigenomic research and enables new discoveries about DNA methylation and its role in male fertility.

Results

Methylomes of sperm and somatic tissues in cattle

We generated single-nucleotide resolution methylation profiles of sperm and somatic cells from three tissues from cattle. The somatic tissues were mammary gland, blood, and brain collected from two cows as biological replicates. Semen was collected twice for each of two bulls, respectively. Through whole-genome bisufite sequencing (WGBS), we obtained considerable data amounts of 2.1 billion unique mapped reads for the three somatic cell types and 1.3 billion unique mapped reads for sperm (Table 1). For each of the 10 samples, 85.5 to 95.6% of the whole cattle genomic CpGs were covered with the average depth from 5.5 to 7.2 × (Table 1). Across the whole genome, CpG dinucleotides were preferentially methylated. Genome wide we saw CpG methylation rate of 72.8 to 78.1% across all samples (Table 1). Bisulfite conversion rates estimated by unmethylated lambda DNA controls supported that we faithfully captured patterns of genomic DNA methylation in these samples (Table 1). Moreover, we detected less than 0.8% non-CG methylation in the non-brain somatic tissues (mammary gland and blood) and sperm cells, in contrast to a higher (~1.3%) non-CG methylation level in the brain samples, which is consistent with previous studies in other species[50].

Global comparisons between sperm methylomes and somatic tissue methylomes

We compared the methylation profiles between pairs of samples at a global CpG level. As expected, the correlations between samples within the same tissue or within sperm were high (r > 0.8) (Figure 1). The correlations between methylation of different tissues were lower, especially the correlation efficiency between sperm and somatic cell methylation which ranged from 0.11 to 0.46 (Figure 1). Cluster analysis according to the CpG methylation also confirmed the consistent results of the biological replicates and reinforced potential methylation differences between somatic cells and sperm cells (Figure S1). PC1 of the PCA analysis explained most of the variances and successfully separated sperm cells from somatic cells (Figure S2). PC2 of the PCA analysis explained most of the variances within somatic cells and successfully separated brain from the other somatic tissues (Figure S2). Moreover, we detected 73,024 differentially methylated cytosine (DMCs) in autosomes between sperm cells and somatic cells (Table S1). These results indicate large differences between sperm and somatic cell methylomes, possibly related to sperm development, in which the genome undergoes a wave of nearly complete demethylation and remethylation.

We next performed a global comparison of distinct genomic features between cattle sperm cells and somatic cells. Both cell types showed high methylation levels for the genic and most of the common repeats, and showed comparably low methylation levels for CpG islands, promoters, low complexity sequence, and tRNA (Figure S3). The satellite was the most variable with significantly lower methylated genome features ($p < 0.01$) in sperms than that in somatic tissues (Figure S3). In contrast, similar methylation levels were seen for all other genomic features between sperm cells and somatic cells. Most of the methylation levels of genomic features showed unimodal patterns of either high or low. Promoter and CpG island showed obvious bimodal patterns which supports their functions in the regulation of gene expression. We also found parts of promoter and CpG island with obviously different methylation levels between sperm and somatic cells (Figure S4). Apart from those, the satellites had largely low to medium methylation levels in sperm cells. Furthermore, the satellites showed globally different methylation patterns between brain (enriched in medium methylation) and the other two somatic tissues (high methylation) (Figure S4).

Different methylation patterns in the partially methylated domains between sperm and somatic cells

To get exact knowledge of the methylation differences between somatic cells and sperm cells, we binned the cattle genome into non-overlapping 20-kb windows. The methylation level of 20-kb windows in sperm was mainly enriched at 80%~100% while in somatic cells, the methylation level distributed more dispersedly and was enriched at 60%~100% (Figure S5a). Although there was no clear indication for bimodal distribution in both somatic and sperm cells, sperm exhibited significantly ($p < 0.01$) more low methylated windows than somatic tissues (~3% vs. 1.2%) when limiting average methylation level to < 50% (Figure S5b, S5c). Moreover, at the chromosome level, obviously more PMDs were seen in the sperm cells than in the somatic cells (Figure S6), e.g. chr7, chr15, chr18, chr21, chr23, and chr29. We identified 69 contiguous PMDs with 47 Mb in length for sperm cells using a hidden Markov model, among which 37 PMDs were supported by at least one kind of somatic tissue (Table S2). However, all the 37 PMDs were derived from brain and only 3 PMDs were from blood samples (Table S2).

We evaluated the enrichment of different genomic features by calculating the ratio (O/E, Observed/Expected) between the observed density in sperm PMDs and the average density in autosomes (Figure S7). The PMD contained fewer genic regions (O/E = 0.36), more CpG island (O/E = 1.74) and more satellite regions which received the highest O/E value of 21.31. A previous study has identified that the satellite enriched pericentromeric regions showed strongly decreased

methylation in human sperm but not in human embryonic stem cells [14]. The localizations of functional bovine pericentromeres are currently unknown but estimated to be near the start of the chromosomes (Figure S6 and Table S2). In our study, we observed clear PMD enrichment (20/69 within the first 3Mb or 35/69 in the first 10% terminal regions of the chromosomes) in cattle sperm cells. Although a few of the PMDs starting from the chromosome start sites were also observed in somatic cells, the interstitial satellite regions showed strongly decreased methylation in sperm cells when compared to somatic cells (Figure 2a, left panel). In the middle of the chromosome, lowly methylated satellite regions contributed to some of the sperm-specific PMDs (Figure 2a, middle panel). The 32 satellite-containing PMDs showed lower methylation levels than the non-satellite-containing PMDs in sperm, which was not seen in the somatic tissues (Figure S8). Moreover, significantly negative correlation ($r = -0.77$, $p = 2.514e-07$) between satellite densities (i.e., total satellite length divided by the region length, Table S2) and methylation levels were seen in sperm cells (Figure 2b). Among somatic tissues, both mammary gland and blood showed significantly positive correlation ($r = 0.59$, $p = 0.00036$; $r = 0.56$, $p = 0.00095$) between satellite densities and methylation levels, while the brain showed no significant correlation ($p = 0.61$) (Figure 2b). Additionally, different methylation patterns in the PMDs appeared in both sperm and somatic cells. For example, the PMD located in the chr29:38860001-39780000 region showed multiple discontinuously HMRs in sperm cells, which was not seen in cells from mammary gland or brain (Figure 2a, right panel).

Function analysis of the genes located in sperm PMDs

There were 168 genes from the refGene database located in the sperm PMD regions. Gene Ontology (GO) analysis showed that they were significantly enriched in the nucleosome and histone-related GO terms, such as: chromosome, DNA binding, nucleus, nucleosome core (Table S3). The genic methylation level in the PMDs of sperm cells was significantly lower ($p<0.01$, student’s t test) than those of somatic tissues (Figure S9). However, the genes seemed to cluster in few PMDs that appeared in both somatic and sperm cells (Table S2). The methylation levels of 11/14 genes related to the histone were commonly hypomethylated (methylation level < 20%) in both somatic and sperm cells (Figure 3a). The histone-related hypomethylated genes, including *HIST1H2AG*, *HIST1H2BN*, *HIST1H1D*, *H2B*, *HIST1H1E*, *HIST1H2BD*, *HIST1H2AC*, *H4*, and *LOC617875*, were clustered in one PMD (chr23:30,700,001-31,700,000, 1Mb) which were, interestingly, also localized in HMRs (Figure 3b). When comparing across sperm cells and somatic tissues, we obtained 28 genes that were significantly less methylated (methylation difference >

20% and FDR <0.01) in sperm cells (Table S4). Two of them (*KIF2B* and *REPIN1*), which are involved in meiosis, were found to be hypomethylated in sperm cells but hypermethylated (methylation level > 80%) in somatic tissues (Figure 3a). *KIF2B* has microtubule depolymerization activity and plays a role in chromosome congression. *REPIN1* is required for initiation of chromosomal DNA replication.

Hypomethylated regions in sperm cells and somatic cells

To identify HMRs for sperm and somatic cells, we used a sliding window approach with a window size of 200 bp and extended the window in 50-bp increments until it contained less than 80% hypomethylated (methylation level < 20%) CpGs. Using this strict threshold, we observed ~ 64k (65.6 Mb in length) HMRs in sperm and ~63k (62.8 Mb in length) HMRs in somatic tissues. Besides the shared 29.8 Mb of HMRs, nearly half of them (~35 Mb in sperm cells and ~33 Mb in somatic cells) were unique to either sperm or somatic cells (Figure 4a). In sperm, all PMDs were supported by 14 ~ 567 HMRs while only 13.5% HMRs were supported by the PMDs. These finding suggested that there were still large portions of HMRs in either sperms or somatic tissues which were not supported by PMDs, in addition to those regions enriched in PMDs.

Based on the O/E values, the promoter, CpG island, and tRNA regions, were most enriched in the HMRs (Figure 4b). Approximately 67% of refGenes had TSS localized in the HMRs of either sperm or somatic cells. Moreover, over half of the CpG islands were overlapped with the promoter regions. This agreed with the long recognized observation that the CpG islands and regions around TSS are generally hypomethylated. However, similar to PMDs as described above, the positive correlation between the methylation differences (between sperm and somatic cells) and the satellite enrichment in sperm cells were still evident, with a 3.29 O/E value in HMRs (Figure 4b). On the other hand, the O/E value of the satellite regions the overlap of somatic tissue-specific HMRs was only 0.23. We also found that 52.4% of the satellite regions were located in the sperm-specific HMRs, while less than 1% of the satellite regions were located in the somatic tissue-specific HMRs.

We found significantly ($p = 1.97 \times 10^{-10}$, student's t test) enrichment of the sperm nucleosomes in sperm HMRs than in somatic tissue HMRs (Table S19). Of the 5,369 nucleosome peaks in the autosomes of the cattle sperm, 35.4 ~ 40.1% were overlapped with sperm HMRs while only 1.5 ~ 3.5% were overlapped with somatic tissue HMRs. Moreover, 71.9% of the nucleosome peaks were overlapped with the shared HMRs among different sperms (Figure S10). The sperm nucleosome

peaks that overlapped with sperm HMRs were mostly (82.5% in length) composed of satellite sequences with high CG density and low gene or promoter content.

Distinct characteristics for the shared HMRs in sperm cells and somatic cells

Most of the TSS were commonly hypomethylated in both sperm and somatic cells. We plotted the average methylation around TSS associated with the common HMRs. Similar with the observation for the HMRs in embryonic stem cells comparing to sperms in the human study [14], we also observed nested HMRs around TSS in somatic tissues when compared to sperm for the common HMRs (Figure 4c). This also was supported by the size distributions of HMRs in sperm cells and somatic cells. The mean size of HMRs was ~729 bp and the median was ~600 bp in sperm cells. In somatic tissues, the mean size of HMRs was ~550 bp and the median was ~450 bp. We then focused on 431 genes that were detected with TSS located in the HMRs of all samples. Over 85.4% of TSS were located in the HMRs of somatic tissues that were nested in at least one side of the sperm HMRs. For example, the extended methylation around the TSS of the CWC15 gene may affect its regulation of pre-mRNA splicing (Figure 4d right panel).

Sperm-specific HMRs were enriched in promoters of genes which were functional in testis

The TSS of 978 genes and 1,275 genes were specifically overlapped with somatic tissue HMRs and sperm HMRs, respectively. Distinct methylation patterns were seen around TSS between sperm cells and somatic tissues (Figure 4c). The genes with TSS overlapping with somatic cell-specific HMRs were significantly enriched in the functional categories related to immunity including: glycoprotein, immunity, innate immunity, and inflammatory response (Table S5). Functional analysis of the genes with TSS specifically overlapping with sperm HMRs illustrated that the genes were related to functions in testis. The most significantly enriched functional category was meiosis (FDR corrected P value = 3.3E-4) (Table S6). Functional annotation clustering analysis also received the highest enrichment score (1.67) for the GO terms related to functions in testis including: DNA methylation involved in gamete generation, piRNA metabolic process, gene silencing by RNA, and male meiosis (Figure S11).

Further validation confirmed the CG methylation status around the TSS for 12 of the 16 genes involved in testis functions, including *BOLL*, *MAEL*, *ASZ1*, *SYCP3*, *CTCFL*, *MND1*, *SPATA22*, *PLD6*, *DDX4*, *RBBP8*, *FKBP6*, and *SYCE1*. The other four genes were false positives caused by the low density of the CG covered around the TSS. Except *CTCFL*, the other 11 genes were detected with co-expression using the program STRING according to previous cattle and mouse

studies (Figure S12). We precisely defined the boundaries of the sperm-specific HMRs overlapping with TSS of the 12 genes (Table S7). Their average methylation levels were significantly higher in sperm cells than the somatic cells (Figure 4e). Moreover, these low methylated regions were strongly enriched for putative binding sites of transcription factors like E2F1, E2F6, and NRF1, which are known to function in testis (Figure S13). We found all 12 genes had CGI-associated hypomethylation. However, the low methylation was not restricted to the CpG island region but extended to a much larger region including repeat elements (Table S7).

We also checked to see if the nucleosome peaks overlap with the sperm specific HMRs in the promoter regions. We did not observe overlaps for the above 12 genes involved in the testis functions but found overlaps for 5 other genes (*TUFT1, WRN, RAB11FIP5, RPS6,* and *HIST1H1C*) related to the GO terms of protein modification and localization. For example, *HIST1H1C* is involved in acetylation, methylation and phosphoprotein. A nucleosome peak was found in the first intron, where low methylation in sperm while high methylation in somatic tissues (Figure 4d left panel).

Hypomethylated BOV-A2 were enriched around the TSS in sperm cells

Most of the repeat elements, especially retrotransposons, showed high methylation levels that are required for transcriptional silencing. Similar to studies in other species, the elements that remain active in cattle, such as LINE/RTE-BovB and LINE/L1, displayed high methylation levels even at high CG density (>= 5%) in both sperm and somatic cells. Moreover, we found that methylation levels in BovB elements negatively correlated with their sequence divergence thus their evolutionary age (Figure S14).

However, there were still some repeats which were hypomethylated (Figure 5a). We extracted the elements that were at least hypomethylated in one sample for LINE, SINE, LTR, DNA, and satellite. The hypomethylated repeats (LINE, SINE, LTR, and DNA) other than satellite were highly enriched within 2 kb of the TSS (Figure 5b). The hypomethylated elements had higher CG density and overlapped or were near at least one CpG island (Figure S15a). The hypomethylated elements had higher levels of DNA methylation variation which implied their potential function in gene expression regulation (Figure S15b). When we checked the age of the hypomethylated elements, we found that only the hypomethylated elements in SINE regions were still associated with young age while the elements in LINE, LTR, and DNA repeat classes were associated with old age (Figure 5c).

We selected the hypomethylated elements with sequence divergence less than 50 in SINE and found 96.8% (675/697) were BOV-A2. The lengths of the young BOV-A2 were enriched between 90-100% of its consensus sequence, supporting that the young age of those hypomethylated BOV-A2. The young BOV-A2 with low methylation may be active, especially near the TSS region which may change the promoter structure by introducing new TFBS. We found 31 genes with hypomethylated BOV-A2 located within 2 kb of their TSS (Table S8). Most of the candidate BOV-A2 showed specifically hypomethylation in sperm cells which illustrated that they may be active in certain developmental stages (Figure 5d). For example, *SYCP3*, one gene functional in spermatogenesis, was found to have a BOV-A2 inserted into an ancient LIME3F element separating it to two parts (Figure 5e). Further searching for transcription factor binding sites (TFBS) in the BOV-A2 sequence found multiple TFBSs and some of them were with function in the testis (Table S9).

Discussion

Using WGBS, this study generated one of the first single-nucleotide resolution cattle sperm DNA methylomes and compared them to the cattle somatic tissue methylomes. The global CG methylation levels detected ranged from 72.8% to 78.1% among our cattle samples, which were similar to those in other mammalian species like humans (~70%), but significantly higher than the earlier RRBS results (approximately 30-40%) [14,47]. It is important to point out that RRBS only reports on the CG-enriched regions of the genome, and the most comprehensive methods like WGBS provides a more representative global estimate. Our genome-wide cattle methylomes confirmed existing knowledge that DNA methylation is important for gene expression and plays a critical role in tissue-specific processes [5,51]. In promoter regions, DNA methylation is associated with transcriptional repression whereas in gene bodies, DNA methylation is generally enriched in the body of highly transcribed genes [52-56]. As reported before for other mammals, global resetting of DNA methylation patterns happens twice during development: once during germ cell development and once during early embryogenesis. Our data permit a genome-wide analysis of the first reprogramming event in cattle.

PMDs: PMDs are large domains of DNA (often greater than 100 kb) that have lower levels of DNA methylation. It was first discovered and defined in cultured human fibroblasts [57]. PMDs were later described in human cancer cells, most mammalian placenta, and mouse germline cells [58-62]. They are often associated with inaccessible chromatin and inactive histone marks, covering entire genes and gene clusters. The mechanisms of PMD formation and the biological significance of PMDs are yet to be determined, but one possibility is that they mark the locations for repressing tissue-specific genes in the inappropriate cell type.

In this study, we found that cattle sperm PMDs share features with those identified in other cell types, especially those identified in mouse germline cells: localization in genomic regions with low GC contents, low CGI density, and low gene density. Thus, we speculate that a similar silencing mechanism may operate in cattle sperm PMDs because they share genomic localizations and structural features with the other PMDs. The existence of PMDs in cattle sperm cells but rarely in the somatic tissues is consistent with our observation that sperm DNA tends to have more hypomethylated CG sites in low GC content regions than in the somatic tissues [62]. In our cattle sperm cells, genes in PMDs commonly included lowly methylated gene clusters related to histone. We also found that genes hypomethylated in sperm but hypermethylated in somatic tissue had testis- or sperm-specific functions. For example, *KIF2B* has microtubule depolymerization activity

and plays a role in chromosome congression. Therefore, HMRs could be greatly involved in the biological process of gene expression.

HMRs: We detected large differences between sperm cells and somatic tissues in terms of HMRs. HMRs often occur in CGIs, however, they also occur outside of CGIs and function as cell-type specific enhancers. As reported before [63-65], the formations of HMRs can be due to 2 possible mechanisms: (1) active transcription and accompanying histone marks like H3K4me3 prevent the access of DNA methyltransferases; and (2) specific protein/DNA complexes, such as CTCF and Sp1, inhibit the methylation machinery in the absence of transcription.

The retained nucleosomes and their post-translational modifications represent potential mediators of epigenetic information transmitted from the sire to its offspring via sperm. In our bovine dataset, the sperm nucleosome peaks that overlapped with sperm HMRs were mostly composed of satellite sequences with high CG density and low gene or promoter content, providing evidence for the predominant retention of sperm nucleosomes in gene deserts.

For shared HMRs, we also observed the "nested" HMR phenomenon as described previously [14], in which the HMRs in sperm cells were larger than those in somatic cells. The function of those genes in our study were significantly enriched in the terms related to epigenetics, like acetylation, phosphoprotein, and mRNA splicing.

For genes overlapped by sperm-specific HMRs, we found the enrichment of GO terms related to male germ cell processes, including DNA methylation involved in gamete generation, piRNA metabolic process, gene silencing by RNA, and male meiosis. We further identified the 12 genes whose TSS overlapped with sperm-specific HMRs (Table S7). For example, the *BOLL* gene belongs to the DAZ gene family required for germ cell development. Loss of this gene function results in azoospermia and male infertility[66]. Acting via the piRNA pathway, genes *ASZ1*, *MAEL*, and *PLD6* play a central role during spermatogenesis by repressing transposable elements and preventing their mobilization, which is essential for germline integrity [66-70]. The *CTCFL* gene is a paralog of *CTCF* and appears to be expressed primarily in the cytoplasm of spermatocytes, whereas *CTCF* is expressed primarily in the nucleus of somatic cells [71]. Although CTCF forms methylation-sensitive insulators that regulate chrX inactivation, the CTFCL protein correlates with resetting of methylation marks during male germ cell differentiation. Genes like *SYCE1* and *SYCP3* encode structural components of the synaptonemal complex, which is involved in synapsis, recombination, and segregation of meiotic chromosomes [72,73]. *MND1* and *SPATA22* encode

proteins required for homologous recombination in meiosis [74,75]. *DDX4*, a DEAD box protein, characterized by the conserved motif Asp-Glu-Ala-Asp (DEAD), encodes a putative RNA helicase, which is specifically expressed in the germ cell lineage in both sexes and functions in germ cell development [76].

Common repeats: In germ cells like sperm, common repeats are normally highly methylated. The conserved piRNA pathway has been proposed to be important for recognizing and silencing repeats in germ cells [77]. However, we still found more than expected HMRs that overlapped common repeats in sperm cells, suggesting some individual elements can evade piRNA-based silencing. Examining patterns of HMR-associated repeats is very informative. One possibility is that just like genes, young repeats contain promoters or regulatory regions and/or their TF binding and transcription activation can facilitate their evading default methylation. Although most of BOV-A2 elements follow the neutral expectation, showing a negative correlation between methylation level and age (represented by their divergence from its consensus sequence), we detected that some BOV-A2 elements were hypomethylated in cattle sperm cells. Similar to the young Alu subfamilies which introduce binding sites for transcription factor SABP in human sperm [78,79], we found some BOV-A2 elements inserted into genes like *SYCP3*, which itself is involved in spermatogenesis. Through examining these Bov-A2 insertions, we found the binding sites for multiple TFs which have functions in testis. As the introduction of TFBS by active Bov-A2 insertions could change the promoter structure, we hypothesize that Bov-A2 insertions in sperm cells may be involved in specific regulation of functional genes. Our results also were consistent with earlier studies, supporting the existence of a system based on environmental and epigenetic signals that is able to spread and mutate the Bov-A2 sequence in the genes expressed during the response to cellular activation signals [80]. Through this adaptive mechanism, ruminants may reinforce and diversify the stress reaction at cellular and individual levels in response to environmental changes.

Centromeric Satellites: In cattle sperm, we found heavy selective hypomethylation of megabase domains of centromeric satellite clusters, as compared to satellites located elsewhere, which were generally methylated at medium levels. Our results also supported the following proposition, in which these regions were initially hypomethylated in male germline cells and then shifted to the hypermethylation status during differentiation into somatic lineage. This agreed with previous observations made in human and mouse [14,81], confirming a conserved epigenetic signature for which the chromosomal centromeric and pericentric regions in male germline cells are specifically

hypomethylated, despite the hypermethylation status in somatic cells. All these observations were consistent with the hypothesis that maintaining hypomethylation of satellites in centromeres might be critical for chromosome segregation during meiosis and their rapid transcriptional activation upon fertilization [82].

In summary, this study provided baseline methylation profiles for cattle sperm and somatic cells at a single-base resolution. We characterized the DNA methylome and assessed DNA methylation patterns. We reported rich data sets of PMDs and HMRs across different tissues and detected a subset of them which correlated with tissue development. Our study contributes to the understanding of cattle DNA methylation patterns and provides foundational information for further investigations.

Materials and methods

Sample collection and DNA isolation

Somatic tissues including parenchymal tissue from the mammary glands, whole blood cells, and frontal cortex of the brain were collected from two healthy adult Holstein cows (3-4 yr old; one lactating and one non-lactating), snap frozen in liquid N_2 immediately after excision, and kept at -80°C until use. Semen straws were collected at twice from two fertile Holstein bulls. Genomic DNA for each tissue was isolated according to the QIAamp DNA Mini Kit protocol (QIAGEN, Valencia, CA, USA). The quality of DNA samples was evaluated using the 2100 Bioanalyzer (Agilent Technologies, Santa Clara, CA, USA) including degradation, and potential RNA contamination, and purity (OD260/OD280), and concentration using a spectrophotometer (NanoDrop Technologies, Rockland, DE) to meet the requirements for library construction.

Library construction and sequencing

The qualified genomic DNA from somatic tissues and sperm were used to construct libraries. Briefly, 3 µg of genomic DNA spiked with unmethylated lambda DNA were fragmented into 200-300 bp using a Covaris S220 (Covaris, Inc., Woburn, MA, USA), followed by teriminal repairing and A-ligation. Different cytosine methylated barcodes were ligated to sonicated DNA for different samples. The DNA bisufite conversion was performed using the EZ DNA Methylation Gold Kit (Zymo Research, Irvine, CA, USA). Then single-stranded DNA fragments were amplified using the KAPA HiFi HotStart Uracil + ReadyMix (2 X) (Kapa Biosystems, Wilmington, MA, USA). The library concentration was quantified using a Qubit 2.0 fluorometer (Life Technologies, Carlsbad, CA, USA) and qPCR (iCycler, BioRad Laboratories, Hercules, CA, USA), and the insert size was checked using the Agilent 2100. To decrease the batch effect, the libraries for one sample were balanced mixed with other libraries with different barcodes and sequenced on different lanes of a HiSeq X Ten (Illumina, San Diego, CA, USA) to generate 150-bp paired-end reads by Novogene (Novogene, Beijing, China).

Sequence alignment and identification of methylcytosine

Programs FastQC (0.11.2) and Trim Galore (0.4.0) were used to generate sequence quality reports and to trim/filter the sequences, respectively (https://www.bioinformatics.babraham.ac.uk/projects/trim_galore/). For each sample, high-quality reads were obtained after trimming low-quality bases and the adapter sequences. The cleaned data for each sample were merged and aligned to the reference genome (*Bos taurus* UMD3.1) using

bowtie2 under the Bismark software (0.14.5) with the parameters -p 3 -N 1 -D 20. The methylcytosine information was extracted using the bismark_methylation_extractor after deduplicating the duplication reads. The first 6 bp were ignored for the paired-end reads to decrease the potential effects of severe bias towards non-methylation in the end-of-reads caused by end repairing.

Global comparison between methylomes of sperm cells and somatic cells

The common CGs with depth over 10× among all sample were used for global comparison between methylomes of sperm cells and somatic cells. Cluster analysis, PCA analysis, and DMC detection were applied using a R package (methykit, R version 3.3.3) [83]. The DMCs were defined as the methylation difference over 30% and q value < 0.01 between sperm cells and somatic cells. The genome structure annotation files for refGene, CpG island, repeats were downloaded from the UCSC database (http://hgdownload.soe.ucsc.edu/goldenPath/). The promoter regions were defined as 1,000 bp around the transcript start sites. The methylation levels for each element in different genomic features were calculated as the average methylation level of the CGs with at least 5× coverage. Only the elements meeting the following criteria were used for further analysis: (1) at least 10% CG detection rate for elements with over 50 CGs; and (2) at least 5 CGs detected for elements with less than 50 CGs. An in-house R script was used to plot the comparison results.

PMD and HMR identification

All the CGs with at least 5× coverage were used for PMD and HMR detection. For PMD detection, we first calculated the average methylation level for each of the non-overlapped 20-kb windows. According to the distribution of the windows' average methylation (Figure S5a), we selected 60% methylation as the threshold to divide the windows after several trails using methylation level at 50%, 55%, 60%, 65% and 70%. The windows with methylation level over 60% were assigned as 1 and the windows with methylation level less than 60% were assigned as 0. Then we applied a hidden Markov model using HMM (one R package) to detect the windows assigned with continuous 0 for each sperm sample. The sperm PMDs used in this study had to meet the following criteria: (1) supported by at least 3 sperm samples; and (2) combined from at least 3 windows.

To identify the contiguous HMRs for sperm cells and somatic cells, we used a sliding window approach with a window size of 200 bp and extended the window by 50-bp steps until it contained less than 80% hypomethylated (methylation level < 20%) CpGs. Only the HMRs with at least 5

CG detected with over 5 × coverage were used for analysis. The GenomicRanges package in R were used to statistics the overlapped HMRs in different tissues or sperms.

Sperm nucleosome loacation detection

Chip-seq data for sperm nucleosome-binding sites detection was downloaded from Gene Expression Omnibus database with the accession number GSM1160360. The sample preparation procedure can be found in [84]. NGSQCToolkit (2.3.3) software was used to filtered the adapters and low quality reads. Then the qualified reads were aligned to the the reference genome (*Bos taurus* UMD3.1) using bowtie2 and peaks were called using MACS (1.4.2) with default parameters. Totally, we detected 5,369 nucleosome location peaks in the autosome of cattle sperm (Table S10).

Gene function analysis

Gene functional annotation analyses were applied using the online DAVID software (https://david.ncifcrf.gov/). Fisher's exact test was used to measure gene enrichment in annotation terms. P-values were corrected by FDR (false discovery rate) to search for significantly enriched terms. The STRING (https://string-db.org/) with default parameters was used to extract co-expressed genes using cattle, and mouse databases because of less supporting data available in the cattle database. We used Homer software [85] to detect enriched motifs within 1,000 bp up and down stream of the TSS for genes involved in spermatogenesis. At the same time, the sequences within 1,000 bp up and down stream of the TSS for refGene were used as background. We used a website software (Genomatrix, https://www.genomatix.de/) to search for TFBS in the Bov-A2 sequence.

Declarations

List of abbreviations

CGI: CpG Island

DMC: differentially methylated cytosine

FAANG: the Functional Annotation of Animal Genome project

FDR: False Discovery Rate

GO: Gene Ontology

HMR: Hypomethylated regions

LINE: long interspersed nuclear element

LTR: Long terminal repeat

PMD: Partially Methylated Domain

O/E: Observed/Expected

RRBS: reduced representation bisulfite sequencing

SINE: short interspersed nuclear element

TFBS: transcription factor binding site

TSS: transcription start site

WGBS: whole genome bisulphite sequencing

Ethics approval

All samples were collected with approval of the US Department of Agriculture (USDA) Agriculture Research Service (ARS) Institutional Animal Care and Use Committee under Protocol 16-016.

Consent to participate

Not applicable

Consent for publication

Not applicable

Availability of data and material

WGBS data are available from GEO under the accession number GSE106538.

Competing interests

All authors declare no potential conflict of interest.

Funding

This work was supported in part by AFRI grant number 2013-67015-20951 from the USDA National Institute of Food and Agriculture (NIFA) Animal Genome and Reproduction Programs and BARD grant number US-4997-17 from the US-Israel Binational Agricultural Research and Development (BARD) Fund.

Authors' contributions

GEL and YZ conceived and designed the experiments. EEC, CL, RLB, and HC collected samples and/or generated NGS data. YZ, DMB, SGS, BDR, and CPVT performed *in silico* prediction and computational analyses. YZ and GEL wrote the paper.

Acknowledgements

We also thank Reuben Anderson, Mary Bowman, Donald Carbaugh, Christina Clover, Sarah McQueeney, Mary Niland, Alexandre Dimtchev, and Research Animal Services for technical assistance. Mention of trade names or commercial products in this article is solely for the purpose of providing specific information and does not imply recommendation or endorsement by the US Department of Agriculture.

Table 1 Whole genome bisufite sequencing of sperm cells (SPERM) and somatic cells from brain (CORTEX), mammary gland (MAM), and blood (WBC) of dairy cattle

Sample	Mapped reads	Bisulfite conversion rate (%)	Methylation level (%)	Average CpG coverage (× fold)	Whole genome CpG covered (%)
CORTEX1	407,336,580	99.31~99.44	74.50	7.03	95
CORTEX2	437,071,564	99.34~99.47	76.30	6.39	86
MAM1	344,055,380	99.38~99.48	73.10	6.19	94
MAM2	393,720,380	99.38~99.44	72.80	7.18	96
WBC1	280,887,870	99.40~99.46	78.10	6.41	94
WBC2	282,543,428	99.25~99.44	77.50	6.83	93
Sperm1 A	296,241,574	99.43~99.52	74.00	5.75	95
Sperm1 B	302,185,176	99.43~99.51	75.70	5.49	93
Sperm2 A	368,996,008	99.40~99.51	76.40	7.10	96
Sperm2 B	289,192,004	99.34~99.41	75.70	5.75	94

References

1. Reik, W., Dean, W. and Walter, J. 2001, Epigenetic reprogramming in mammalian development. *Science*, **293**, 1089-1093.
2. Morgan, H. D., Santos, F., Green, K., Dean, W. and Reik, W. 2005, Epigenetic reprogramming in mammals. *Human molecular genetics*, **14**, R47-R58.
3. Sasaki, H. and Matsui, Y. 2008, Epigenetic events in mammalian germ-cell development: reprogramming and beyond. *Nature Reviews Genetics*, **9**, 129-140.
4. Igarashi, J., Muroi, S., Kawashima, H., et al. 2008, Quantitative analysis of human tissue-specific differences in methylation. *Biochemical and biophysical research communications*, **376**, 658-664.
5. Law, J. A. and Jacobsen, S. E. 2010, Establishing, maintaining and modifying DNA methylation patterns in plants and animals. *Nature Reviews Genetics*, **11**, 204-220.
6. Robertson, K. D. 2005, DNA methylation and human disease. *Nature Reviews Genetics*, **6**, 597-610.
7. Noonepalle, S. K. R., Lee, E. J., Ouzounova, M., et al. 2015, Promoter methylation regulates interferon-γ induced indoleamine 2, 3-dioxygenase expression in breast cancer. *Cancer research*, **75**, 4060-4060.
8. Walsh, C. P., Chaillet, J. R. and Bestor, T. H. 1998, Transcription of IAP endogenous retroviruses is constrained by cytosine methylation. *Nature genetics*, **20**, 116-117.
9. Hajkova, P., Erhardt, S., Lane, N., et al. 2002, Epigenetic reprogramming in mouse primordial germ cells. *Mech Dev*, **117**, 15-23.
10. Dean, W., Santos, F. and Reik, W. 2003, Epigenetic reprogramming in early mammalian development and following somatic nuclear transfer. *Semin Cell Dev Biol*, **14**, 93-100.
11. Sasaki, H. and Matsui, Y. 2008, Epigenetic events in mammalian germ-cell development: reprogramming and beyond. *Nat Rev Genet*, **9**, 129-140.
12. Hammoud, S. S., Nix, D. A., Zhang, H., Purwar, J., Carrell, D. T. and Cairns, B. R. 2009, Distinctive chromatin in human sperm packages genes for embryo development. *Nature*, **460**, 473-478.
13. Popp, C., Dean, W., Feng, S., et al. 2010, Genome-wide erasure of DNA methylation in mouse primordial germ cells is affected by AID deficiency. *Nature*, **463**, 1101-1105.
14. Molaro, A., Hodges, E., Fang, F., et al. 2011, Sperm methylation profiles reveal features of epigenetic inheritance and evolution in primates. *Cell*, **146**, 1029-1041.
15. Zhang, X., Yazaki, J., Sundaresan, A., et al. 2006, Genome-wide high-resolution mapping and functional analysis of DNA methylation in Arabidopsis. *Cell*, **126**, 1189-1201.
16. Cokus, S. J., Feng, S., Zhang, X., et al. 2008, Shotgun bisulphite sequencing of the Arabidopsis genome reveals DNA methylation patterning. *Nature*, **452**, 215-219.
17. Lister, R., Pelizzola, M., Dowen, R. H., et al. 2009, Human DNA methylomes at base resolution show widespread epigenomic differences. *Nature*, **462**, 315-322.
18. Xie, W., Barr, C. L., Kim, A., et al. 2012, Base-resolution analyses of sequence and parent-of-origin dependent DNA methylation in the mouse genome. *Cell*, **148**, 816-831.
19. Habibi, E., Brinkman, A. B., Arand, J., et al. 2013, Whole-genome bisulfite sequencing of two distinct interconvertible DNA methylomes of mouse embryonic stem cells. *Cell stem cell*, **13**, 360-369.
20. Schultz, M. D., He, Y., Whitaker, J. W., et al. 2015, Human body epigenome maps reveal noncanonical DNA methylation variation. *Nature*.
21. Bourc'his, D. and Bestor, T. H. 2004, Meiotic catastrophe and retrotransposon reactivation in male germ cells lacking Dnmt3L. *Nature*, **431**, 96-99.

22. Oakes, C. C., La Salle, S., Smiraglia, D. J., Robaire, B. and Trasler, J. M. 2007, Developmental acquisition of genome-wide DNA methylation occurs prior to meiosis in male germ cells. *Dev Biol*, **307**, 368-379.
23. Benchaib, M., Braun, V., Ressnikof, D., et al. 2005, Influence of global sperm DNA methylation on IVF results. *Hum Reprod*, **20**, 768-773.
24. Houshdaran, S., Cortessis, V. K., Siegmund, K., Yang, A., Laird, P. W. and Sokol, R. Z. 2007, Widespread epigenetic abnormalities suggest a broad DNA methylation erasure defect in abnormal human sperm. *PLoS One*, **2**, e1289.
25. Nanassy, L. and Carrell, D. T. 2008, Paternal effects on early embryogenesis. *J Exp Clin Assist Reprod*, **5**, 2.
26. Khazamipour, N., Noruzinia, M., Fatehmanesh, P., Keyhanee, M. and Pujol, P. 2009, MTHFR promoter hypermethylation in testicular biopsies of patients with non-obstructive azoospermia: the role of epigenetics in male infertility. *Hum Reprod*, **24**, 2361-2364.
27. Navarro-Costa, P., Nogueira, P., Carvalho, M., et al. 2010, Incorrect DNA methylation of the DAZL promoter CpG island associates with defective human sperm. *Hum Reprod*, **25**, 2647-2654.
28. Wu, W., Shen, O., Qin, Y., et al. 2010, Idiopathic male infertility is strongly associated with aberrant promoter methylation of methylenetetrahydrofolate reductase (MTHFR). *PLoS One*, **5**, e13884.
29. Pacheco, S. E., Houseman, E. A., Christensen, B. C., et al. 2011, Integrative DNA methylation and gene expression analyses identify DNA packaging and epigenetic regulatory genes associated with low motility sperm. *PLoS One*, **6**, e20280.
30. Adelson, D. L., Raison, J. M. and Edgar, R. C. 2009, Characterization and distribution of retrotransposons and simple sequence repeats in the bovine genome. *Proceedings of the National Academy of Sciences*, **106**, 12855-12860.
31. Iwasaki, Y. W., Siomi, M. C. and Siomi, H. 2015, PIWI-Interacting RNA: Its Biogenesis and Functions. *Annual review of biochemistry*, **84**, 405-433.
32. Czech, B. and Hannon, G. J. 2016, One Loop to Rule Them All: The Ping-Pong Cycle and piRNA-Guided Silencing. *Trends in biochemical sciences*, **41**, 324-337.
33. Bakshi, A., Herke, S. W., Batzer, M. A. and Kim, J. 2016, DNA methylation variation of human-specific Alu repeats. *Epigenetics*, **11**, 163-173.
34. Taiwo, O., Wilson, G. A., Morris, T., et al. 2012, Methylome analysis using MeDIP-seq with low DNA concentrations. *Nature protocols*, **7**, 617-636.
35. de Montera, B., Fournier, E., Shojaei Saadi, H. A., et al. 2013, Combined methylation mapping of 5mC and 5hmC during early embryonic stages in bovine. *BMC Genomics*, **14**, 406.
36. Couldrey, C., Brauning, R., Bracegirdle, J., Maclean, P., Henderson, H. V. and McEwan, J. C. 2014, Genome-wide DNA methylation patterns and transcription analysis in sheep muscle.
37. Gao, F., Zhang, J., Jiang, P., et al. 2014, Marked methylation changes in intestinal genes during the perinatal period of preterm neonates. *BMC genomics*, **15**, 716.
38. Huang, Y.-Z., Sun, J.-J., Zhang, L.-Z., et al. 2014, Genome-wide DNA methylation profiles and their relationships with mRNA and the microRNA transcriptome in bovine muscle tissue (Bos taurine). *Scientific reports*, **4**.
39. Lee, J.-R., Hong, C. P., Moon, J.-W., et al. 2014, Genome-wide analysis of DNA methylation patterns in horse. *BMC genomics*, **15**, 598.
40. Su, J., Wang, Y., Xing, X., Liu, J. and Zhang, Y. 2014, Genome-wide analysis of DNA methylation in bovine placentas. *BMC genomics*, **15**, 12.

41. Shojaei Saadi, H. A., O'Doherty, A. M., Gagne, D., et al. 2014, An integrated platform for bovine DNA methylome analysis suitable for small samples. *BMC Genomics*, **15**, 451.
42. Cao, J., Wei, C., Liu, D., et al. 2015, DNA methylation Landscape of body size variation in sheep. *Scientific reports*, **5**.
43. Choi, M., Lee, J., Le, M. T., et al. 2015, Genome-wide analysis of DNA methylation in pigs using reduced representation bisulfite sequencing. *DNA Research*, **22**, 343-355.
44. Schachtschneider, K. M., Madsen, O., Park, C., Rund, L. A., Groenen, M. A. and Schook, L. B. 2015, Adult porcine genome-wide DNA methylation patterns support pigs as a biomedical model. *BMC genomics*, **16**, 743.
45. Salilew-Wondim, D., Fournier, E., Hoelker, M., et al. 2015, Genome-Wide DNA Methylation Patterns of Bovine Blastocysts Developed In Vivo from Embryos Completed Different Stages of Development In Vitro. *PLoS One*, **10**, e0140467.
46. Schroeder, D. I., Jayashankar, K., Douglas, K. C., et al. 2015, Early Developmental and Evolutionary Origins of Gene Body DNA Methylation Patterns in Mammalian Placentas. *PLoS Genet*, **11**, e1005442.
47. Zhou, Y., Xu, L., Bickhart, D. M., et al. 2016, Reduced representation bisulphite sequencing of ten bovine somatic tissues reveals DNA methylation patterns and their impacts on gene expression. *BMC Genomics*, **17**, 779.
48. Kropp, J., Carrillo, J. A., Namous, H., et al. 2017, Male fertility status is associated with DNA methylation signatures in sperm and transcriptomic profiles of bovine preimplantation embryos. *BMC Genomics*, **18**, 280.
49. Andersson, L., Archibald, A. L., Bottema, C. D., et al. 2015, Coordinated international action to accelerate genome-to-phenome with FAANG, the Functional Annotation of Animal Genomes project. *Genome biology*, **16**, 57.
50. Lister, R., Mukamel, E. A., Nery, J. R., et al. 2013, Global epigenomic reconfiguration during mammalian brain development. *Science*, **341**, 1237905.
51. Wilkinson, M. F. 2015, Evidence that DNA methylation engenders dynamic gene regulation. *Proceedings of the National Academy of Sciences*, 201502195.
52. Jones, P. A. 1999, The DNA methylation paradox. *Trends Genet*, **15**, 34-37.
53. Lorincz, M. C., Dickerson, D. R., Schmitt, M. and Groudine, M. 2004, Intragenic DNA methylation alters chromatin structure and elongation efficiency in mammalian cells. *Nature structural & molecular biology*, **11**, 1068-1075.
54. Ball, M. P., Li, J. B., Gao, Y., et al. 2009, Targeted and genome-scale strategies reveal gene-body methylation signatures in human cells. *Nature biotechnology*, **27**, 361-368.
55. Laurent, L., Wong, E., Li, G., et al. 2010, Dynamic changes in the human methylome during differentiation. *Genome Res*, **20**, 320-331.
56. Varley, K. E., Gertz, J., Bowling, K. M., et al. 2013, Dynamic DNA methylation across diverse human cell lines and tissues. *Genome research*, **23**, 555-567.
57. Lister, R., Pelizzola, M., Dowen, R. H., et al. 2009, Human DNA methylomes at base resolution show widespread epigenomic differences. *Nature*, **462**, 315-322.
58. Berman, B. P., Weisenberger, D. J., Aman, J. F., et al. 2011, Regions of focal DNA hypermethylation and long-range hypomethylation in colorectal cancer coincide with nuclear lamina-associated domains. *Nature genetics*, **44**, 40-46.
59. Hon, G. C., Hawkins, R. D., Caballero, O. L., et al. 2012, Global DNA hypomethylation coupled to repressive chromatin domain formation and gene silencing in breast cancer. *Genome Res*, **22**, 246-258.
60. Schroeder, D. I., Blair, J. D., Lott, P., Yu, H. O., Hong, D. and Crary, F. 2013, The human placenta methylome. *Proc Natl Acad Sci U S A*, **110**.

61. Schroeder, D. I., Jayashankar, K., Douglas, K. C., et al. 2015, Early developmental and evolutionary origins of gene body DNA methylation patterns in mammalian placentas. *PLoS Genet*, **11**.

62. Kubo, N., Toh, H., Shirane, K., et al. 2015, DNA methylation and gene expression dynamics during spermatogonial stem cell differentiation in the early postnatal mouse testis. *BMC Genomics*, **16**, 624.

63. Gaszner, M. and Felsenfeld, G. 2006, Insulators: exploiting transcriptional and epigenetic mechanisms. *Nat Rev Genet*, **7**, 703-713.

64. Dhayalan, A., Rajavelu, A., Rathert, P., et al. 2010, The Dnmt3a PWWP domain reads histone 3 lysine 36 trimethylation and guides DNA methylation. *The Journal of biological chemistry*, **285**, 26114-26120.

65. Zhang, Y., Jurkowska, R., Soeroes, S., et al. 2010, Chromatin methylation activity of Dnmt3a and Dnmt3a/3L is guided by interaction of the ADD domain with the histone H3 tail. *Nucleic Acids Res*, **38**, 4246-4253.

66. Kostova, E., Yeung, C. H., Luetjens, C. M., Brune, M., Nieschlag, E. and Gromoll, J. 2007, Association of three isoforms of the meiotic BOULE gene with spermatogenic failure in infertile men. *Molecular human reproduction*, **13**, 85-93.

67. Yan, W., Rajkovic, A., Viveiros, M. M., Burns, K. H., Eppig, J. J. and Matzuk, M. M. 2002, Identification of Gasz, an evolutionarily conserved gene expressed exclusively in germ cells and encoding a protein with four ankyrin repeats, a sterile-alpha motif, and a basic leucine zipper. *Molecular endocrinology (Baltimore, Md.)*, **16**, 1168-1184.

68. Huang, H., Gao, Q., Peng, X., et al. 2011, piRNA-associated germline nuage formation and spermatogenesis require MitoPLD profusogenic mitochondrial-surface lipid signaling. *Developmental cell*, **20**, 376-387.

69. Wang, Q., Liu, X., Tang, N., et al. 2013, GASZ promotes germ cell derivation from embryonic stem cells. *Stem cell research*, **11**, 845-860.

70. Li, X. D., Zhang, J. X., Jiang, L. J., et al. 2016, Overexpression of maelstrom promotes bladder urothelial carcinoma cell aggressiveness by epigenetically downregulating MTSS1 through DNMT3B. *Oncogene*, **35**, 6281-6292.

71. Loukinov, D. I., Pugacheva, E., Vatolin, S., et al. 2002, BORIS, a novel male germ-line-specific protein associated with epigenetic reprogramming events, shares the same 11-zinc-finger domain with CTCF, the insulator protein involved in reading imprinting marks in the soma. *Proc Natl Acad Sci U S A*, **99**, 6806-6811.

72. Fraune, J., Brochier-Armanet, C., Alsheimer, M. and Benavente, R. 2013, Phylogenies of central element proteins reveal the dynamic evolutionary history of the mammalian synaptonemal complex: ancient and recent components. *Genetics*, **195**, 781-793.

73. Syrjanen, J. L., Pellegrini, L. and Davies, O. R. 2014, A molecular model for the role of SYCP3 in meiotic chromosome organisation. *eLife*, **3**.

74. Chi, P., San Filippo, J., Sehorn, M. G., Petukhova, G. V. and Sung, P. 2007, Bipartite stimulatory action of the Hop2-Mnd1 complex on the Rad51 recombinase. *Genes Dev*, **21**, 1747-1757.

75. Dorosh, A., Tepla, O., Zatecka, E., Ded, L., Koci, K. and Peknicova, J. 2013, Expression analysis of MND1/GAJ, SPATA22, GAPDHS and ACR genes in testicular biopsies from non-obstructive azoospermia (NOA) patients. *Reproductive biology and endocrinology : RB&E*, **11**, 42.

76. Tilgner, K., Atkinson, S. P., Yung, S., et al. 2010, Expression of GFP under the control of the RNA helicase VASA permits fluorescence-activated cell sorting isolation of human primordial germ cells. *Stem cells (Dayton, Ohio)*, **28**, 84-92.

77. Aravin, A. A. and Hannon, G. J. 2008, Small RNA silencing pathways in germ and stem cells. *Cold Spring Harbor symposia on quantitative biology*, **73**, 283-290.
78. Kochanek, S., Renz, D. and Doerfler, W. 1993, DNA methylation in the Alu sequences of diploid and haploid primary human cells. *The EMBO journal*, **12**, 1141-1151.
79. Chesnokov, I. N. and Schmid, C. W. 1995, Specific Alu binding protein from human sperm chromatin prevents DNA methylation. *The Journal of biological chemistry*, **270**, 18539-18542.
80. Damiani, G., Florio, S., Panelli, S., Capelli, E. and Cuccia, M. 2008, The Bov-A2 retroelement played a crucial role in the evolution of ruminants. *Rivista di biologia*, **101**, 375-404.
81. Yamagata, K., Yamazaki, T., Miki, H., et al. 2007, Centromeric DNA hypomethylation as an epigenetic signature discriminates between germ and somatic cell lineages. *Dev Biol*, **312**, 419-426.
82. Probst, A. V., Okamoto, I., Casanova, M., El Marjou, F., Le Baccon, P. and Almouzni, G. 2010, A strand-specific burst in transcription of pericentric satellites is required for chromocenter formation and early mouse development. *Developmental cell*, **19**, 625-638.
83. Akalin, A., Kormaksson, M., Li, S., et al. 2012, methylKit: a comprehensive R package for the analysis of genome-wide DNA methylation profiles. *Genome Biol*, **13**, R87.
84. Samans, B., Yang, Y., Krebs, S., et al. 2014, Uniformity of nucleosome preservation pattern in Mammalian sperm and its connection to repetitive DNA elements. *Developmental cell*, **30**, 23-35.
85. Heinz, S., Benner, C., Spann, N., et al. 2010, Simple combinations of lineage-determining transcription factors prime cis-regulatory elements required for macrophage and B cell identities. *Molecular cell*, **38**, 576-589.

Figure Legends

Figure 1. Correlation analysis between each sample using common CpGs. Sperm1 A and B: sperm samples from Holstein 1; Sperm2 A and B: sperm samples from Holstein 2; WBC: whole blood cells; MAM: mammary glands; CORTEX: frontal cortex of the brain.

Figure 2. Characteristics of the sperm cell PMDs. (a). CpG methylation status of the PMDs using chr29 as an example. PMDs are indicated by the dashed lines. (b). Correlation analysis between satellite density and methylation levels of PMDs in sperm cells and somatic cells. Sperm1 A and B: sperm samples from Holstein 1; Sperm2 A and B: sperm samples from Holstein 2; WBC: whole blood cells; MAM: mammary glands; CORTEX: frontal cortex of the brain.

Figure 3. Methylation levels of the genes located within the sperm PMDs. (a) Dot plot of the methylation levels of the genes located in the sperm PMDs. Only gene methylation level with standard deviations less than 20% among somatic tissues or sperm cells were used for plotting. (b) CpG methylation status of the partial PMD (chr23:30,700,001-31,700,000) clustered genes related to histones. Sperm1 A and B: sperm samples from Holstein 1; Sperm2 A and B: sperm samples from Holstein 2; WBC: whole blood cells; MAM: mammary glands; CORTEX: frontal cortex of the brain.

Figure 4. Comparison of HMRs between sperm and somatic cells. (a) Venn plot for the HMRs between sperm and somatic cells. (b) Genomic feature enrichment analysis in the HMRs that are shared or unique for sperm and somatic cells. (c) UP: methylation distribution around the TSS in the shared HMRs between sperm and somatic cells. DOWN: CpG methylation status of two genes for the nested HMRs around TSS. (d) Methylation distribution around the TSS in the sperm- or somatic cell- specific HMRs. (e) Methylation levels of the genes with TSS located in the sperm-specific HMRs in sperm and somatic cells. Sperm1 A and B: sperm samples from Holstein 1; Sperm2 A and B: sperm samples from Holstein 2; WBC: whole blood cells; MAM: mammary glands; CORTEX: frontal cortex of the brain.

Figure 5. Analysis of the hypomethylated repeats. (a) Percentage of hypomethylated elements for common repeats. (b) Enrichment of the hypomethylated repeats around TSS. (c) Sequence divergence and thus age distribution of common repeats (x-axis: % substitutions in matching region compared to the consensus). (d) Heat map plot for the methylation levels of hypomethylated BOV-A2 in sperm and somatic cells. Each row represents one BOV-A2 element. (e) An example of the BOV-A2 element inserted in the region around TSS near the *SYCP3* gene. Sperm1 A and B:

sperm samples from Holstein 1; Sperm2 A and B: sperm samples from Holstein 2; WBC: whole blood cells; MAM: mammary glands; CORTEX: frontal cortex of the brain.

Figure

Click here to download Figure Figures.pdf

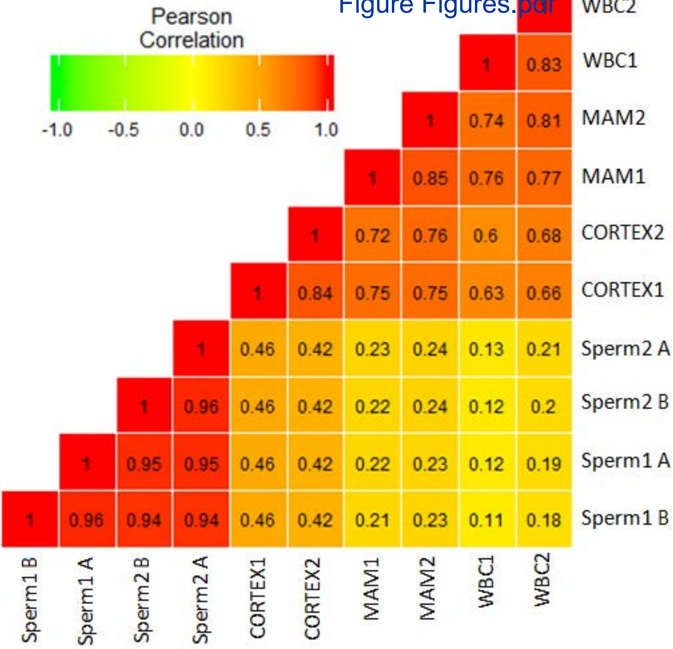

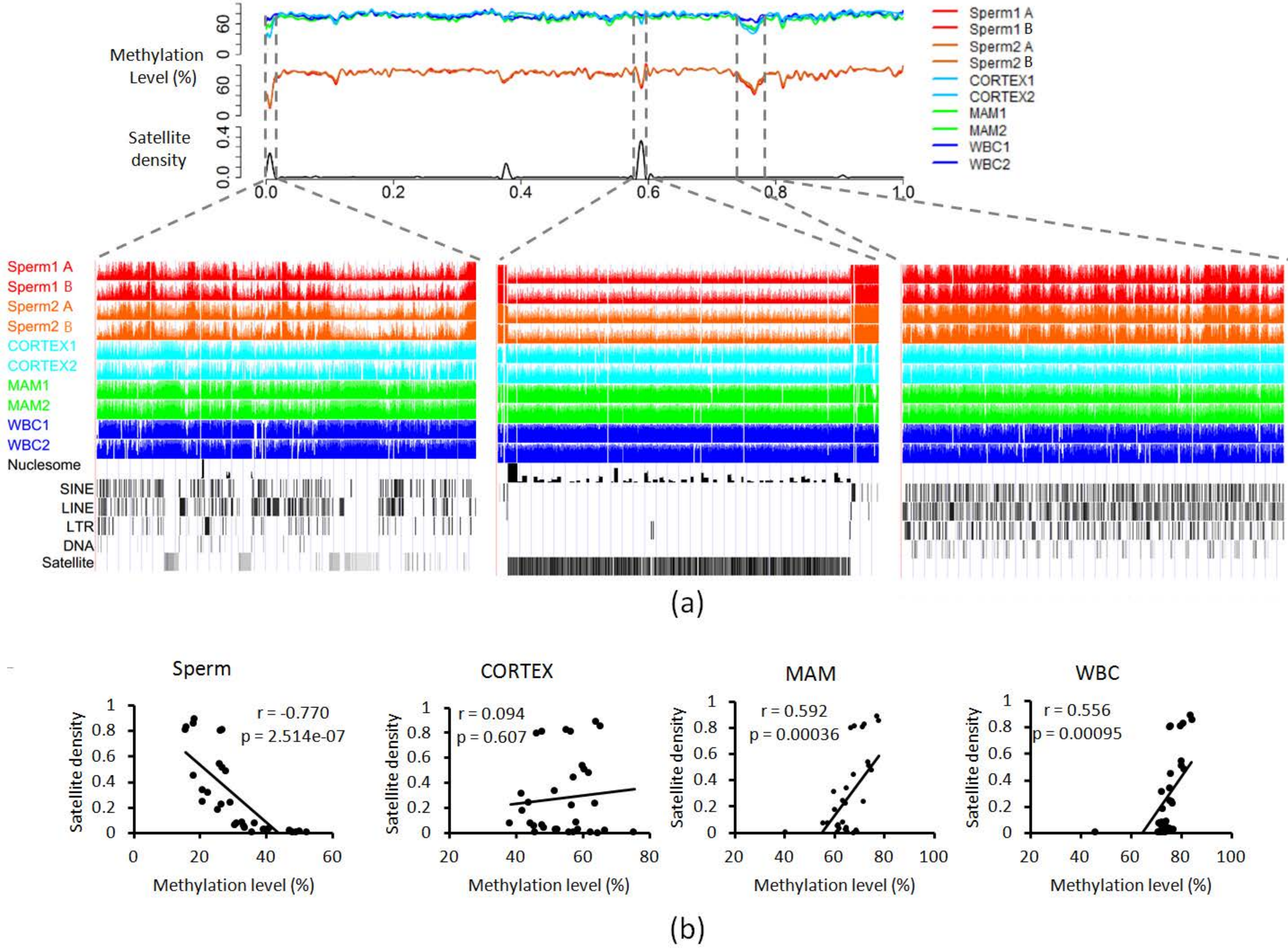
Methylation Level (%)
Satellite density
Sperm1 A
Sperm1 B
Sperm2 A
Sperm2 B
CORTEX1
CORTEX2
MAM1
MAM2
WBC1
WBC2
Nucleosome
SINE
LINE
LTR
DNA
Satellite
(a)
Sperm
r = -0.770
p = 2.514e-07
CORTEX
r = 0.094
p = 0.607
MAM
r = 0.592
p = 0.00036
WBC
r = 0.556
p = 0.00095
Satellite density
Methylation level (%)
(b)

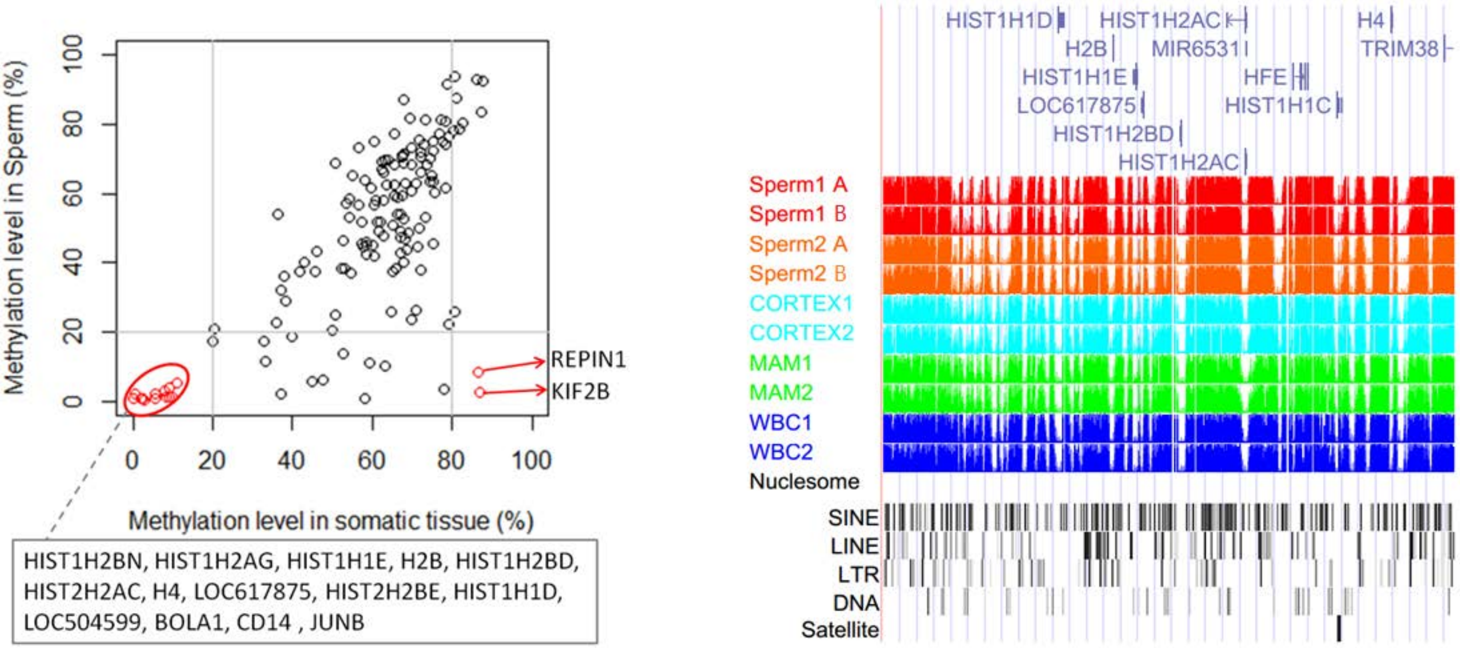

Methylation level in Sperm (%)
0
20
40
60
80
100
Methylation level in somatic tissue (%)
REPIN1
KIF2B
HIST1H2BN, HIST1H2AG, HIST1H1E, H2B, HIST1H2BD, HIST2H2AC, H4, LOC617875, HIST2H2BE, HIST1H1D, LOC504599, BOLA1, CD14 , JUNB
HIST1H1D
HIST1H2AC
H4
H2B
MIR6531
TRIM38
HIST1H1E
HFE
LOC617875
HIST1H1C
HIST1H2BD
HIST1H2AC
Sperm1 A
Sperm1 B
Sperm2 A
Sperm2 B
CORTEX1
CORTEX2
MAM1
MAM2
WBC1
WBC2
Nucleosome
SINE
LINE
LTR
DNA
Satellite

(a) (b)

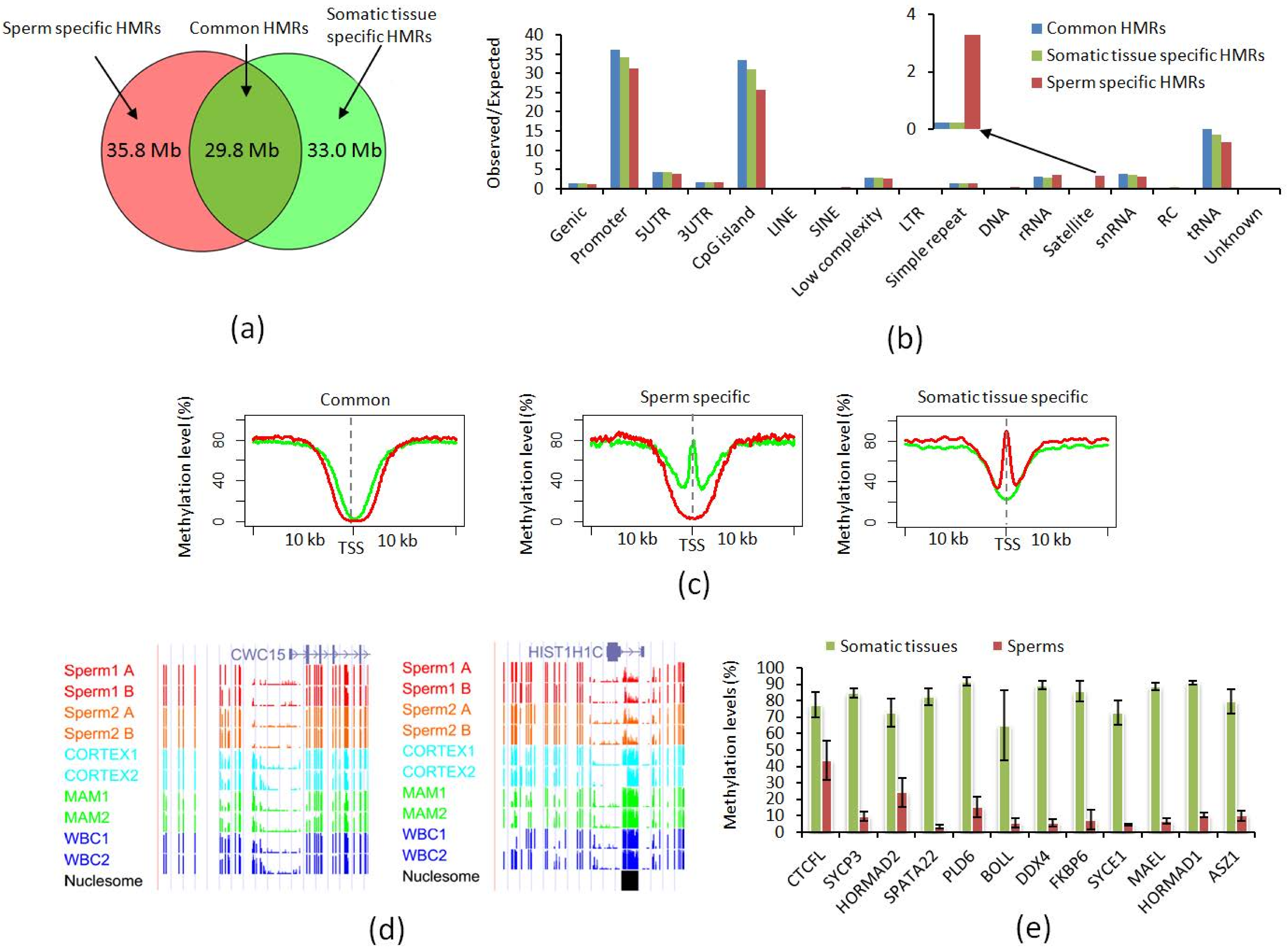

Sperm specific HMRs
Common HMRs
Somatic tissue specific HMRs
35.8 Mb
29.8 Mb
33.0 Mb
(a)
Observed/Expected
Common HMRs
Somatic tissue specific HMRs
Sperm specific HMRs
Genic
Promoter
5UTR
3UTR
CpG island
LINE
SINE
Low complexity
LTR
Simple repeat
DNA
rRNA
Satellite
snRNA
RC
tRNA
Unknown
(b)
Common
Sperm specific
Somatic tissue specific
Methylation level (%)
10 kb
TSS
10 kb
(c)
CWC15
HIST1H1C
Sperm1 A
Sperm1 B
Sperm2 A
Sperm2 B
CORTEX1
CORTEX2
MAM1
MAM2
WBC1
WBC2
Nucleosome
(d)
Somatic tissues
Sperms
Methylation levels (%)
CTCFL
SYCP3
HORMAD2
SPATA22
PLD6
BOLL
DDX4
FKBP6
SYCE1
MAEL
HORMAD1
ASZ1
(e)

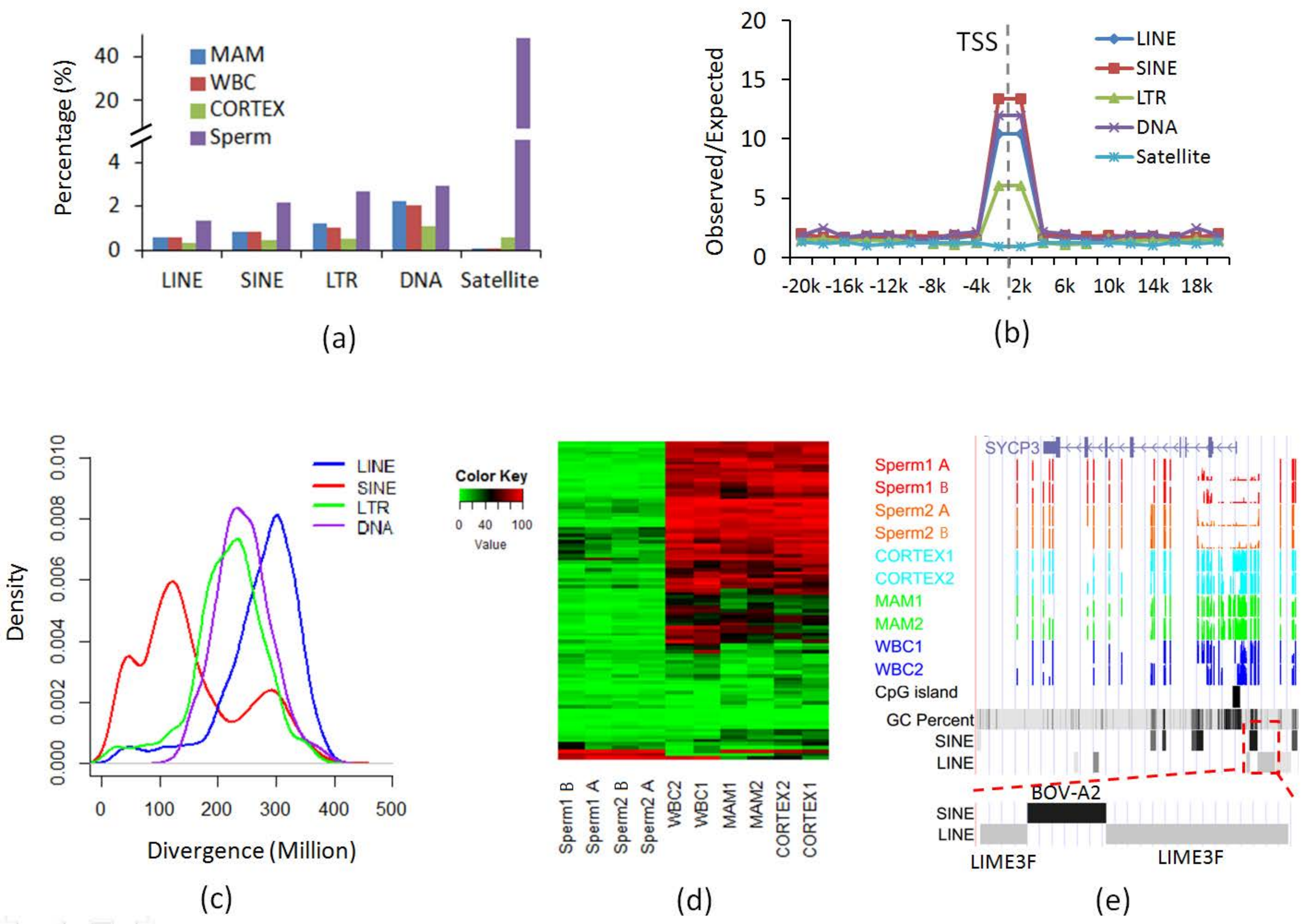

MAM
WBC
CORTEX
Sperm
Percentage (%)
40
20
4
2
0
LINE
SINE
LTR
DNA
Satellite
(a)
TSS
LINE
SINE
LTR
DNA
Satellite
Observed/Expected
20
15
10
5
0
-20k -16k -12k -8k -4k 2k 6k 10k 14k 18k
(b)
LINE
SINE
LTR
DNA
Density
0.010
0.008
0.006
0.004
0.002
0.000
0 100 200 300 400 500
Divergence (Million)
(c)
Color Key
0 40 100
Value
Sperm1 B
Sperm1 A
Sperm2 B
Sperm2 A
WBC2
WBC1
MAM1
MAM2
CORTEX2
CORTEX1
(d)
SYCP3
Sperm1 A
Sperm1 B
Sperm2 A
Sperm2 B
CORTEX1
CORTEX2
MAM1
MAM2
WBC1
WBC2
CpG island
GC Percent
SINE
LINE
BOV-A2
SINE
LINE
LIME3F
LIME3F
(e)

Click here to access/download
Supplementary Material
Supp_Tab_v8.xlsx

Supplementary Material

Click here to access/download
Supplementary Material
Supp_Fig_v8.docx
