# Supplementary material for: Comparative whole genome DNA methylation profiling of cattle sperm and somatic tissues reveals striking hypomethylated patterns in sperm
Source: Gigascience. 2018 Apr 10;7(5):giy039. doi: 10.1093/gigascience/giy039 (PMC5928411; doi:10.1093/gigascience/giy039)
Supplement: Supplemental material [file giy039_supp.zip › Supp_Fig_v9.docx]

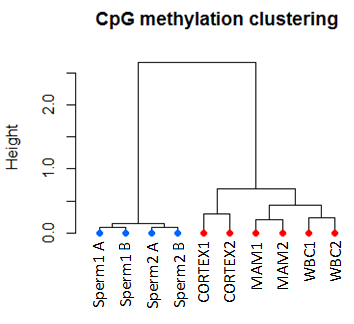


**Figure S1. Cluster analysis according to the CpG methylation.** Cluster analysis according to the CpG methylation confirmed the consistent results of the biological replicates and reinforced potential methylation differences between somatic cells and sperm cells.


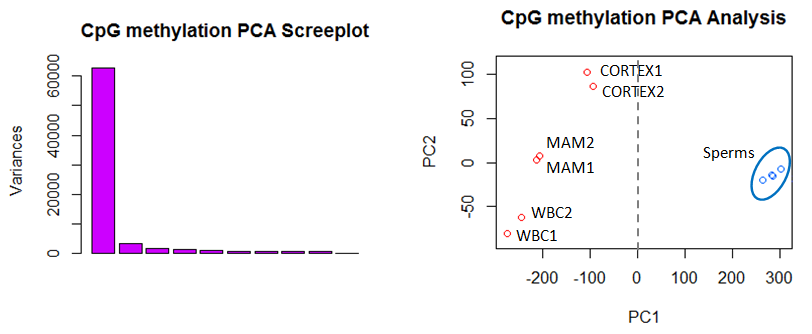


**Figure S2. Principal component analysis based on CpG methylation.** PC1 of the PCA analysis explained most of the variances and successfully separated sperm cells from somatic cells. PC2 of the PCA analysis explained most of the variances within somatic cells and successfully separated brain from the other somatic tissues.


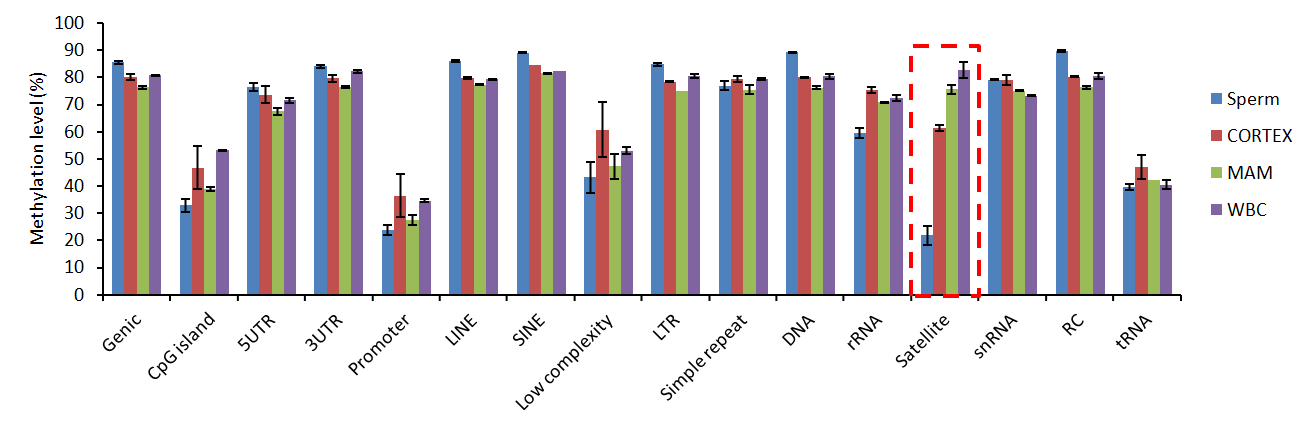


**Figure S3. Global comparisons of distinct genomic features between sperm cells and somatic cells in cattle.** Both sperm cells and somatic cells showed high methylation levels for the genic and most of the common repeats, and showed comparably low methylation levels for CpG island, promoter, low complexity and tRNA regions. Satellite was the most variable with significantly lower methylated genome features (p < 0.01) in sperm cells compared to somatic cells. In contrast, similar methylation levels were seen for all other genomic features between sperm and somatic cells. We calculated the CpG island methylation level after the repeat region was masked.


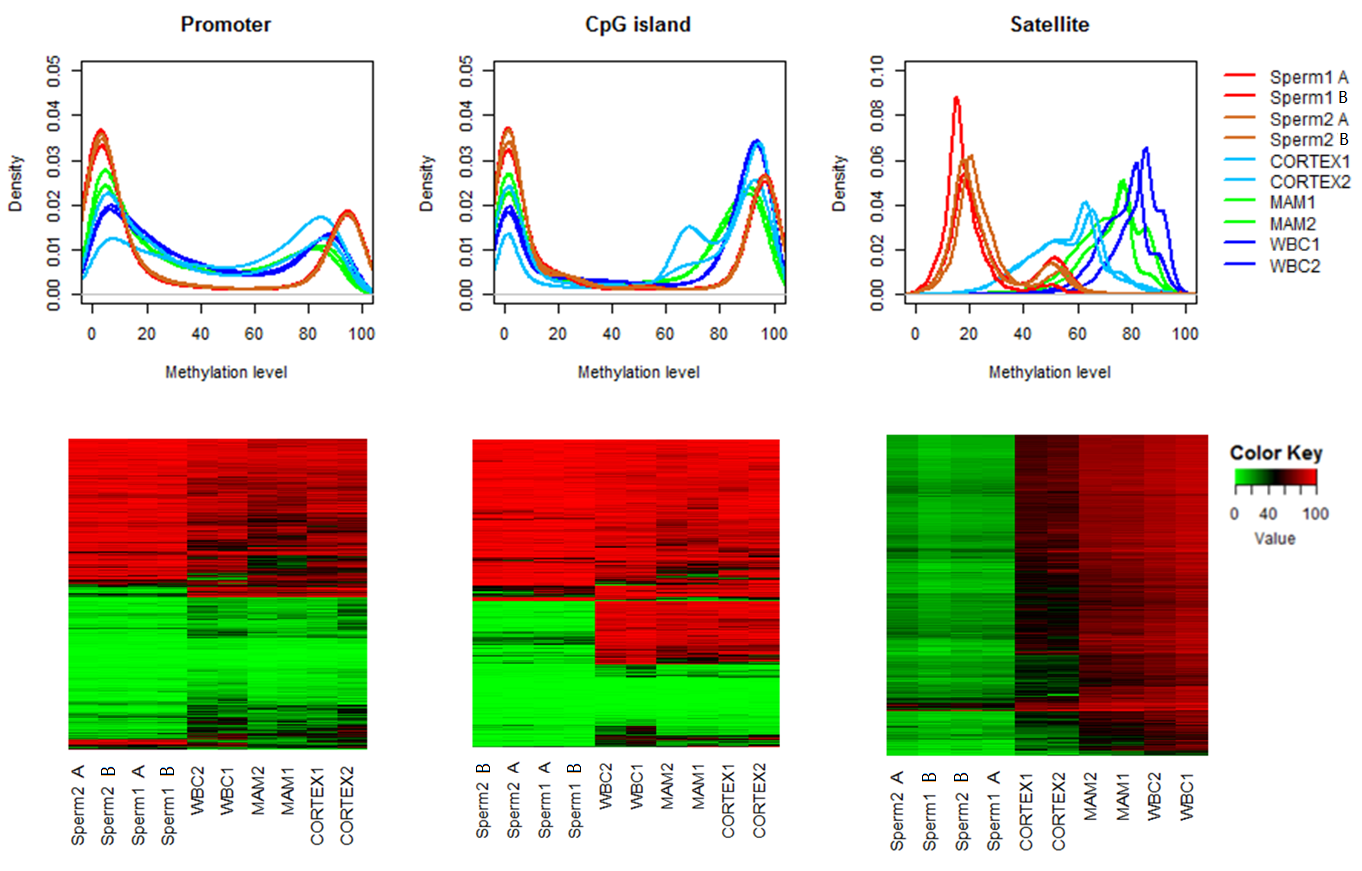


**Figure S4. Methylation level distribution histograms and heat map plots for selected genomic features.** Most of the methylation levels of genomic features showed a unimodal pattern of either high or low. Promoter and CpG island showed obvious bimodal patterns which supports their functions in the regulation of gene expression. We also found parts of promoter and CpG island with obviously different methylation levels between sperm cells and somatic cells. Apart from those, the satellites had largely low to medium methylation levels in sperm cells. Furthermore, the satellite showed globally different methylation patterns between brain and the other two somatic tissues. The brain exhibited globally medium methylation in satellite while the satellites of other two somatic tissues were enriched in high methylation.

**
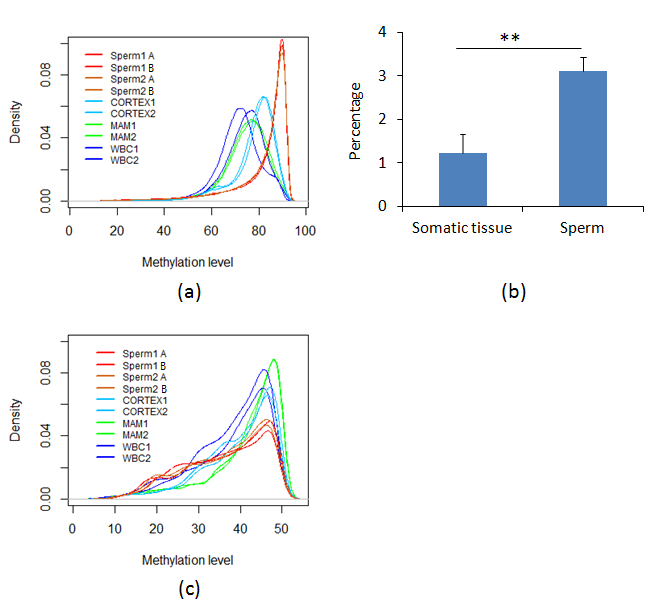
**

**Figure S5. Methylation level distribution histograms for sperm cells and somatic cells.** (**a**) The methylation level of 20-kb windows in sperm was mainly enriched in 80%~100% while in somatic tissues, the methylation level distributed more dispersedly and enriched in 60%~100%. (**b** and **c**) Although there was no clear indication for bimodal distribution in both somatic cells and sperm, sperm cells had significantly (p < 0.01) more low methylated windows than somatic tissues (~3% vs. 1.2%) when zooming in for average methylation level < 25%.


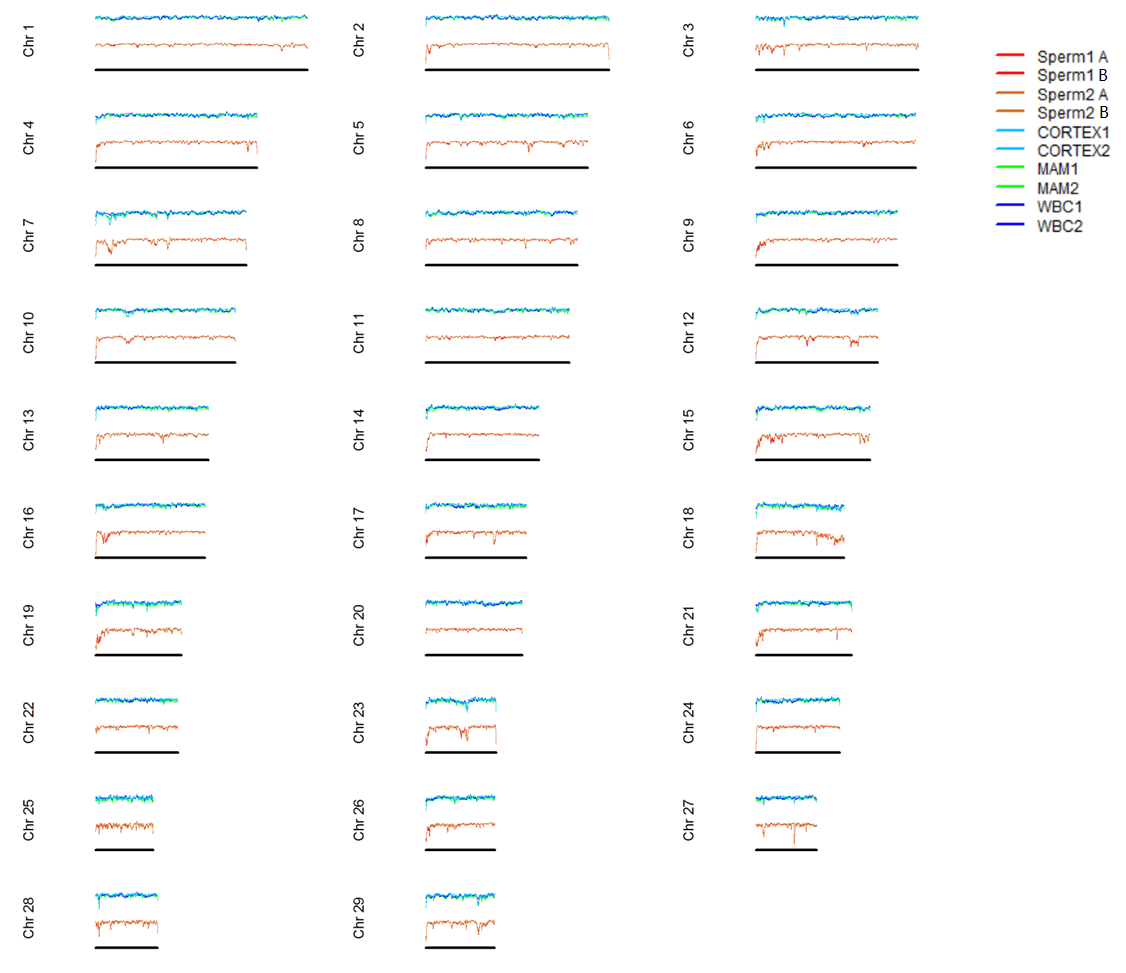


**Figure S6. Distribution of partially methylated domains (PMDs) within the cattle genome.** We identified 69 contiguous PMDs with 47 Mb in length for sperm cells using a hidden Markov model, among which 37 PMDs were supported by at least one kind of somatic cell. At the chromosome level, more PMDs were seen in the sperm cells than in the somatic cells, e.g. chr7, chr15, chr18, chr21, chr23, and chr29. The cattle assembly is represented as black bars with relative DNA methylation levels indicated by color lines above on the chromosomes.


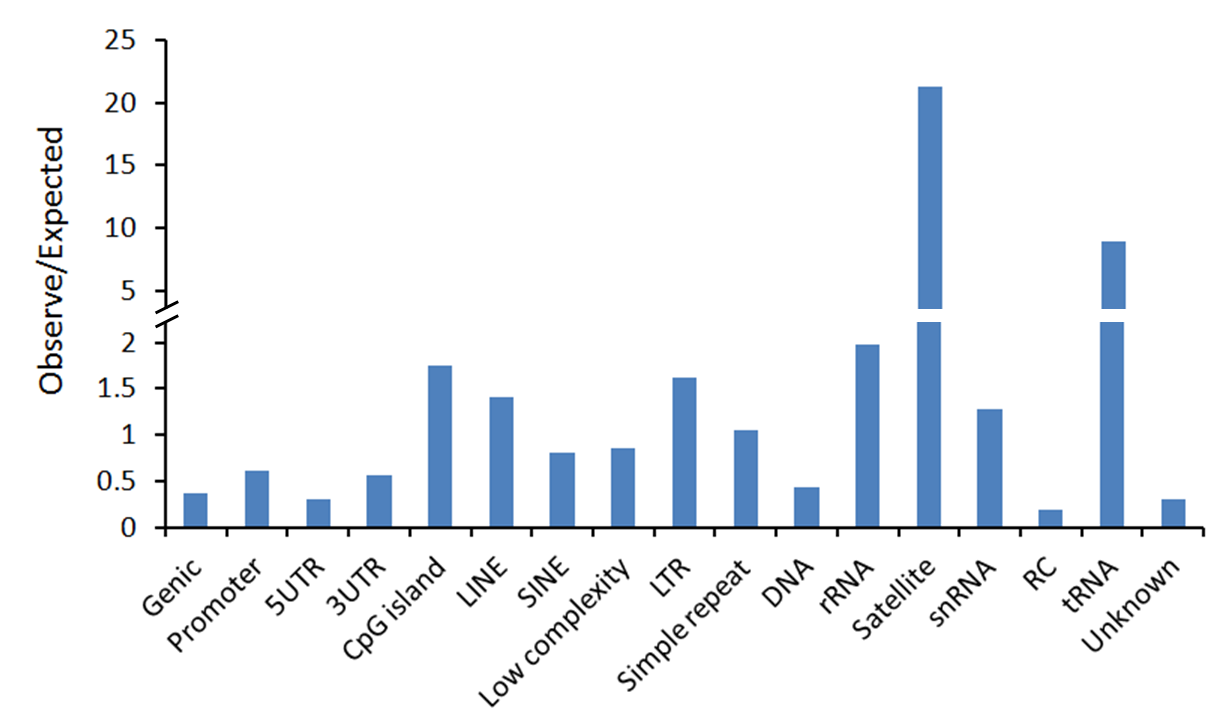


**Figure S7. Enrichment of different genomic features by calculating the Observed/Expected ratio between the observed density in sperm-specific PMDs and the average density in autosomes.** The PMD contained less genic regions (O/E =0.36), more CpG island (O/E =1.74), and more satellite which received the highest O/E value of 21.31.


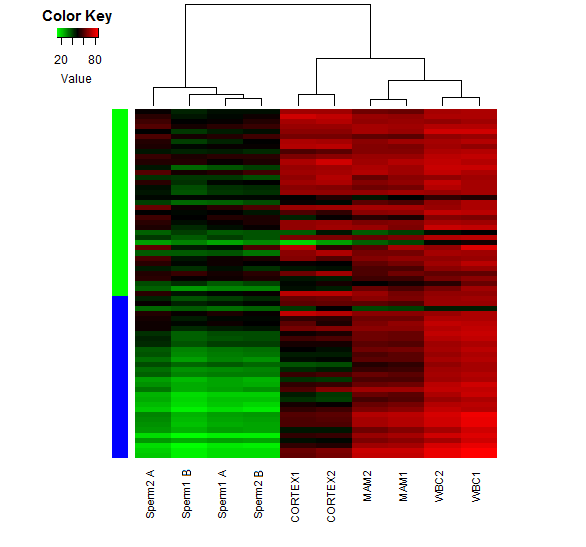


**Figure S8. Heat map plot for the methylation levels of satellite-containing PMDs in sperm and somatic cells.** The 32 satellite-containing PMDs (labelled by blue bar on the left) showed lower methylation levels than the non-satellite-containing PMDs (labelled by green bar on the left) in sperm cells, which was not seen in the somatic cells.


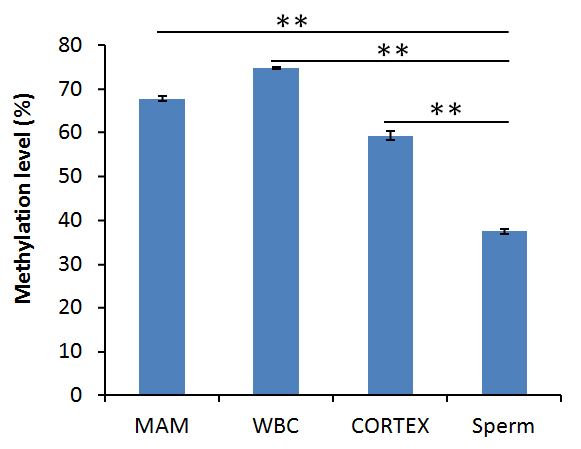


**Figure S9.** **The genic methylation level in the PMD of sperm cells was significantly lower (p<0.01, student’s t test) than those of somatic cells.**


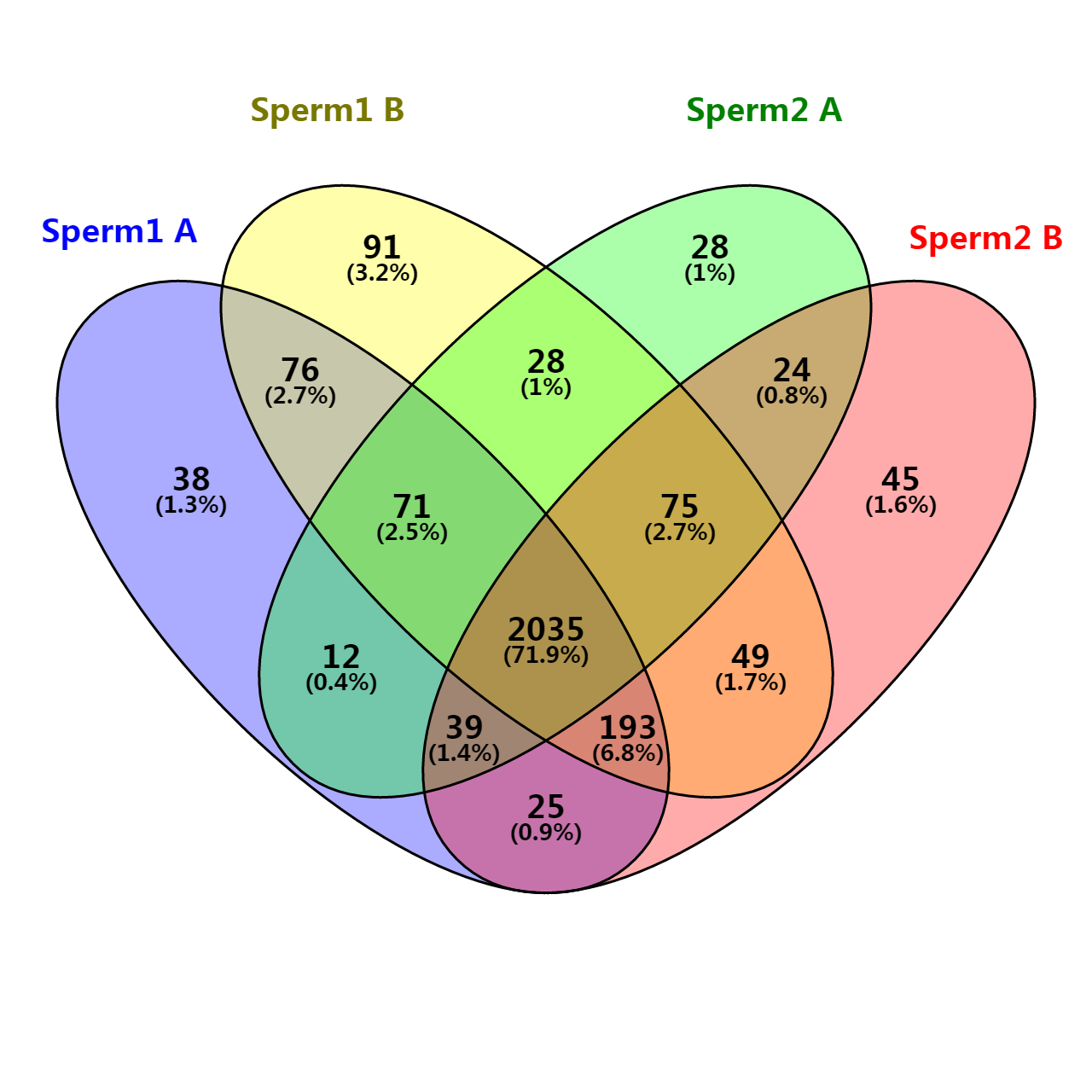


**Figure S10. Venn plot for overlaps of HMR-associated nucleosomes among four sperm samples.** 2035 out of 2829 (~71.9%) nucleosome peaks were overlapped with the shared HMRs among different sperm samples.
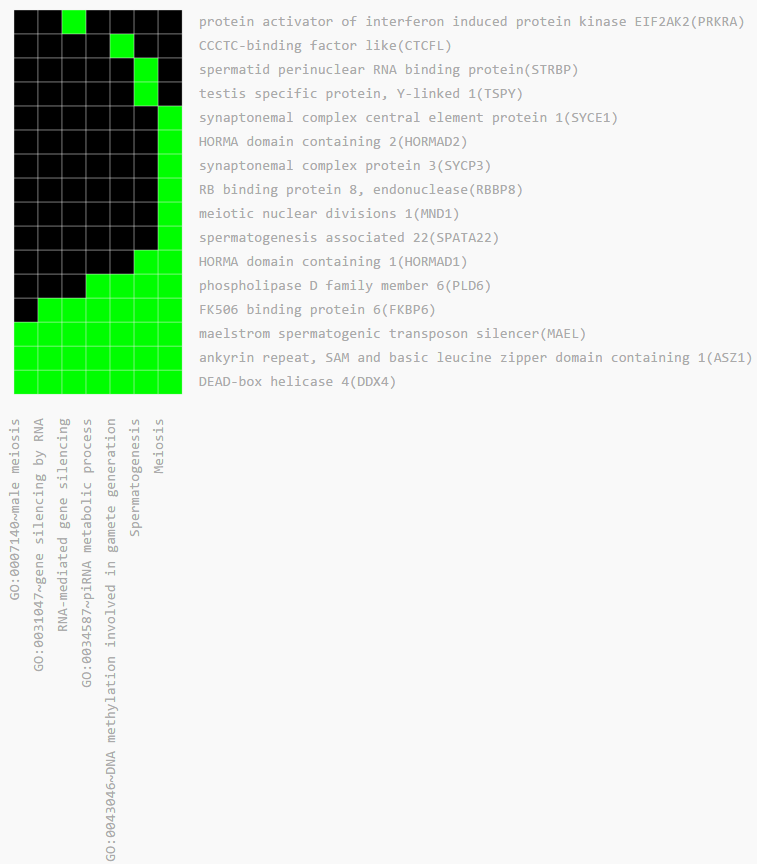


**Figure S11. Functional annotation clustering analysis.** Functional analysis for the genes with TSS specifically overlapped with sperm HMRs illustrated that the genes were related to functions in testis. GO terms related to functions in testis received the highest enrichment score (1.67) including: DNA methylation involved in gamete generation, piRNA metabolic process, gene silencing by RNA, and male meiosis. Green: gene-term association positively reported; Black: gene-term association not reported yet.


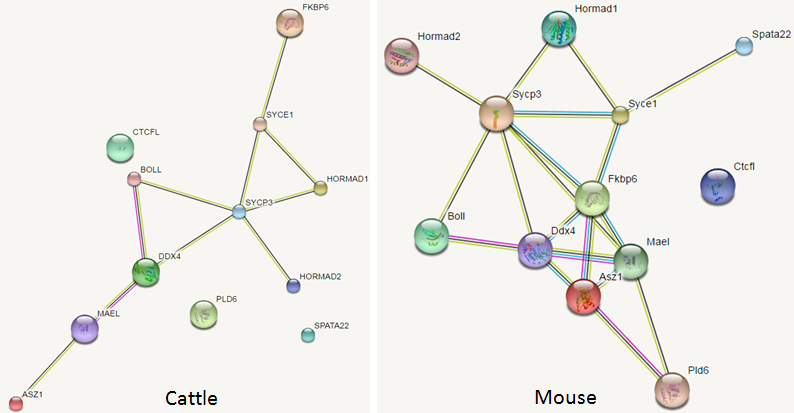


**Figure S12**. **Functional analysis for the genes with TSS specifically overlapped with sperm HMRs illustrated that the genes were related to functions in testis.** Functional analysis confirmed the CG methylation status around the TSS for 12 of the 16 genes involved in the functions of the testis. The other four genes as the false positive were caused by the low density of the CG coverage around the TSS. Except *CECFL*, the other 11 genes were detected with co-expression according to previous cattle and mouse studies.


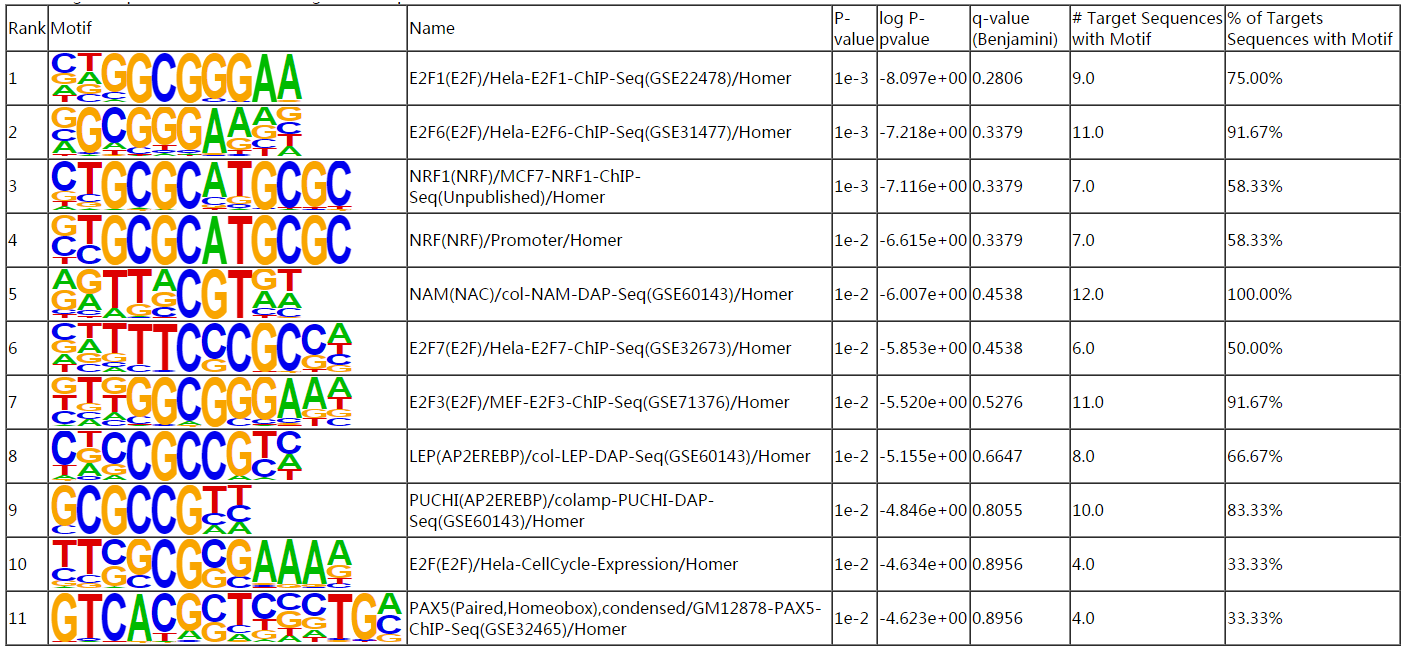


**Figure S13. Sperm-specific HMRs were enriched for transcription factor binding sites known to function in the testis.** We precisely defined the boundaries of the sperm-specific HMRs overlapped with TSS of the 12 genes (Table S7). Their average methylation levels were significantly higher in sperm cells than the somatic cells (Figure 4e). Moreover, those low methylated regions were strongly enriched for putative binding sites of transcription factors like E2F1, E2F6, NRF1, which are known to function in testis.


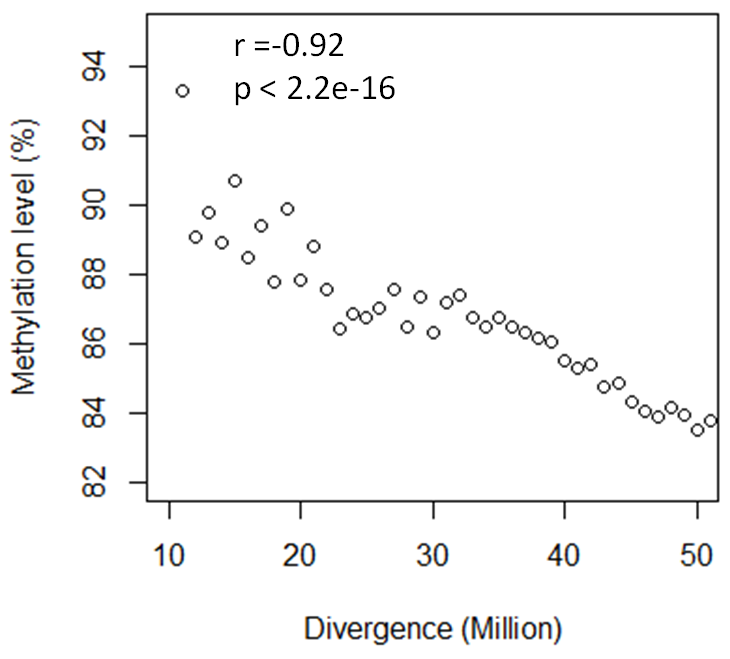


**Figure S14. Methylation levels in BovB elements negatively correlated with their divergence thus their evolutionary age.** Most of the repeat elements, especially retrotransposons, showed high methylation levels that are required for transcriptional silencing. Similar to studies in other species, the elements that remain active in cattle, such as LINE/RTE-BovB and LINE/L1, displayed high methylation levels even at high CG density (>= 5%) in both sperm cells and somatic cells. X-axis: % substitutions in matching region compared to the consensus


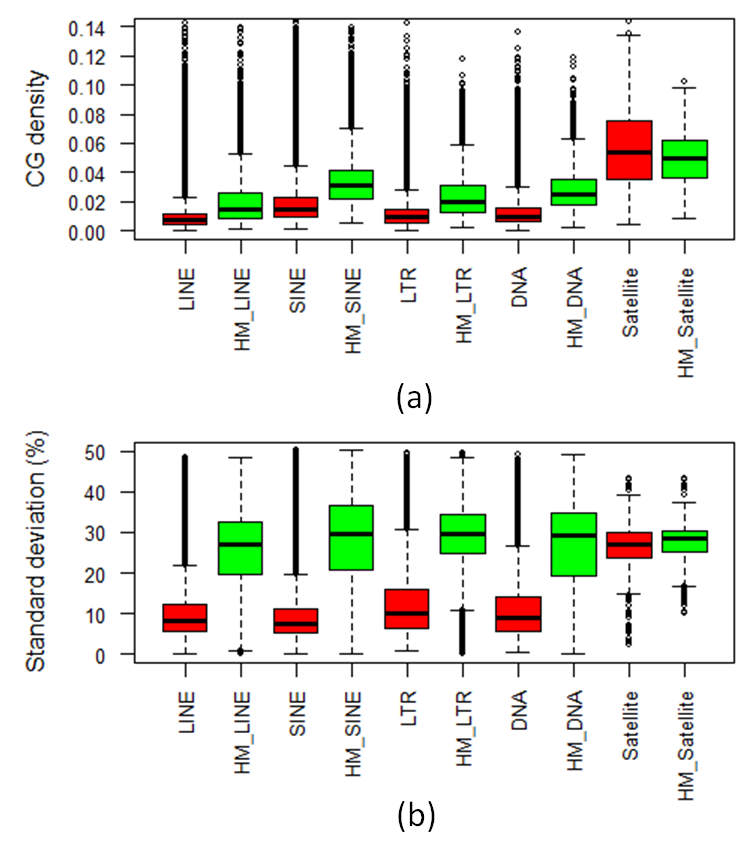


**Figure S15.** **The hypomethylated elements had higher CG density and overlapped or were near at least one CpG island (a). The hypomethylated elements were with higher levels of DNA methylation variation which implies their potential function in gene expression regulation (b).**
