# Supplementary material for: Comparative whole genome DNA methylation profiling of cattle sperm and somatic tissues reveals striking hypomethylated patterns in sperm
Source: Gigascience. 2018 Apr 10;7(5):giy039. doi: 10.1093/gigascience/giy039 (PMC5928411; doi:10.1093/gigascience/giy039)
Supplement: Response_to_Reviewer_Comments_Original_Submission.pdf [file giy039_response_to_reviewer_comments_original_submission.pdf]

Author's Response To Reviewer Comments

Close

GIGA-D-17-00303
Genome-wide sequencing and comparative profiling of cattle sperm DNA methylome reveals its hypomethylated patterns Yang Zhou; Erin Connor; Derek Bickhart; Congjun Li; Ransom Baldwin; Steven Schroeder; Benjamin Rosen; Liguo Yang; Curtis Van Tassell; George Liu GigaScience

Dear Dr. Liu,

Your manuscript "Genome-wide sequencing and comparative profiling of cattle sperm DNA methylome reveals its hypomethylated patterns" (GIGA-D-17-00303) has been assessed by our reviewers. Based on these reports, and my own assessment as Editor, it is potentially acceptable for publication in GigaScience, if you are able to convincingly address the reviewers' main concerns.

In particular, two main points raised by the reviewers seem crucial to me:

1) Reviewer 1 points out that the standard in the field, also endorsed by ENCODE communities, is a 10x coverage to call methylation differences, whereas in many of your analyses you are calling differences with 5x coverage. In Gigascience articles we wish to showcase examples of best practice in big data science, therefore this is a major concern for us. Are you able to add more data, as suggested by the reviewer? The second option the reviewer brings up (combining two replicates for comparison) seems sub-optimal, but if this is the only feasible way to address the coverage issue, we can discuss in light of your revision whether this approach may be sufficient to deal with the concern.
AU: Thanks for your consideration. Unfortunately, we could not add more data due to sample and financial constraints. By looking up and comparing with other publications, especially Ziller et al. and other 3 published papers (See our answer to Reviewer 1’s Q1), we are confident that the quality of this work (results and data analyses) is on a par with these studies appearing on other high profiling journals.

2) The use of "brain tissue" in your experiments (without further specification) seems not precise enough to describe the tissue. Please specify which precise brain region(s) have been used, as mentioned by reviewer 2.
AU: The brain tissue was from prefrontal cortex.

Reviewer 2 also noted that the GEO accessions are not live yet - in case of acceptance of a revised manuscript, the data needs to be publicly available prior to publication.
AU: Once the paper is accepted, we will make sure the GEO accession is publicly available.

The reviewers' full reports are below.

Once you have made the necessary corrections, please submit a revised manuscript online at:

https://giga.editorialmanager.com/

If you have forgotten your username or password please use the "Send Login Details" link to get your login information. For security reasons, your password will be reset.

Please include a point-by-point within the 'Response to Reviewers' box in the submission system. Please ensure you describe additional experiments that were carried out and include a detailed rebuttal of any criticisms or requested revisions that you disagreed with. Please also ensure that your revised manuscript conforms to the journal style, which can be found in the Instructions for Authors on the journal homepage.

The due date for submitting the revised version of your article is 23 Apr 2018.

We look forward to receiving your revised manuscript soon.

Best wishes,

Hans Zauner
GigaScience
www.gigasciencejournal.com

Reviewer reports:
Reviewer #1: Zhou et al. use WGBS sequencing to assess the differences in methylations patterns between bovine sperm and somatic cells. They observe distinct differences in methylation levels between sperm which e.g. relate to genes involved in spermatogenesis and meiosis related processes. In general the paper is sound and well written.

I only have two comments which I think the authors should deal with, but one which in my opinion is essential for the paper.

Major comment:
R1Q1. 1) The minimum coverage (5x) used to call methylation differences for many of the analysis is too low. WGBS, as other next generation sequencing methodologies, have high levels of stochastic variation thus a minimum of 5x will lead to a large number of false positive calls. Instead a minimum of at least 10x must be used, which is the consensus in the methylation community and also recommended by the different ENCODE communities. The most straightforward way to achieve this is by sequencing deeper, however I can imagine that this may not be an option due to financial constraints. An other option is to combine the two biological replicated followed by doing the analysis with a threshold of 10x and compare these results to the ones obtained by 5x to test the level of biases.
AU: In our manuscript, we always used at least 10x coverage to call methylation differences at single CG site level. This is consistent with the recommendation by the research communities. Only when we did the analysis at the region level (PMD, HMR or elements), we used the CG sites with over 5x coverage to calculate the methylation level, for which Reviewer 1 was concerned.
When we designed our sequencing strategy, we decided to sequence each of two replicates to 18X, instead of sequencing one sample to 30X. It was based on Ziller et al.'s conclusions: "For DMR identification using the sample types analyzed in this study, we recommend per-sample coverage in the range of 5–15×, depending on the magnitude of methylation differences between the groups and whether a smoothing or single CpG–based DMR identification strategy is used." "If the goal, however, is primarily to identify long DMRs with large methylation differences, we find that reducing coverage down to 1× or 2× per sample is acceptable." "the findings strongly argue for the use of at least two separate biological replicates for DMR analysis." (Ziller et al, 2014). Actually, for methylation analysis at region level, lower cutoffs (like 5x) had been routinely used, which is because that all 5× CG sites detected in one region across multiple samples will be simultaneously considered. Thus, our statistical power is greatly enhanced, even in the presence of stochastic

variation of WGBS or NGS. Merging them is not necessarily a good practice, because by removing the variances between the replicates, we will lose this power.
This common practice was supported by multiple published papers, (1) In Zachary Smith et al., "For 100-bp tiles, reads for all the CpGs that were covered more than fivefold within the tile were pooled and used to estimate the methylation level as described for single CpGs" (Zachary et al., 2014); (2) In Matthias Farlik et al., "We further removed regions that had not been covered in at least three samples and regions that had not been covered with at least three reads in at least one sample" (Matthias et al., 2016); (3) in Isabel Mendizabal et al., "We considered 18 889 743 CpG sites that were covered by at least five reads in all 10 tissues" (Isabel et al., 2015). In our manuscript, we used similar if not more strict thresholds by considering the number of CGs in genomic regions/elements, and included the replicates to calculate the methylation level. For example, we considered the PMDs supported by at least 3 out of 4 sperm samples as the true ones. In the HMR and element analysis, only the HMRs and elements meeting the following criteria were used for further analysis: (1) at least 10% CG detection rate for regions/elements with over 50 CGs; and (2) at least 5 CGs detected for regions/elements with less than 50 CGs. Finally, our results were generally consistent and supported with the previous human and mouse studies.

References
1. Smith, Z. D.; Chan, M. M.; Humm, K. C.; Karnik, R.; Mekhoubad, S.; Regev, A.; Eggan, K.; Meissner, A., DNA methylation dynamics of the human preimplantation embryo. Nature 2014, 511 (7511), 611.
2. Ziller M. J.; Hansen K. D.; Meissner A.; Aryee M. J. Coverage recommendations for methylation analysis by whole-genome bisulfite sequencing. Nat Methods. 2015, 12(3):230-2.
3. Mendizabal, I.; Yi, S. V., Whole-genome bisulfite sequencing maps from multiple human tissues reveal novel CpG islands associated with tissue-specific regulation. Human molecular genetics 2015, 25 (1), 69-82.
4. Farlik, M.; Halbritter, F.; Müller, F.; Choudry, F. A.; Ebert, P.; Klughammer, J.; Farrow, S.; Santoro, A.; Ciaurro, V.; Mathur, A., DNA methylation dynamics of human hematopoietic stem cell differentiation. Cell stem cell 2016, 19 (6), 808-822.

Minor comment:
R1Q2. 1) The title is somewhat cryptic to me. The authors should try to make it more clear. Something in the this direction:
Comparative whole genome DNA methylome profiling of cattle sperm and somatic tissues reveals sticking hypomethylated patterns in sperm.
AU: It was changed into “Comparative whole genome DNA methylome profiling of cattle sperm and somatic tissues reveals striking hypomethylated patterns in sperm”.

Reviewer #2:

Q1. There are minor grammar issues throughout the manuscript, please check the grammar.
AU: We did that.
Q2. Line 48. Brain is listed as a tissue, please define the precise brain region used in this study.
AU: The brain tissue is from prefrontal cortex.
Q3. Line 122. The Figure description for Figure S1 contains track changes. Please correct the figure legend.
AU: We fixed it.
Q4. Line 124. You refer to Figure S2 and state "successfully separated brain". However, for Figure S2, brain is labeled as cortex. Please specify which brain cortex is used. Please make this change throughout the manuscript and supplemental materials.
AU: The brain tissue was from prefrontal cortex. We fixed it throughout the manuscript and

supplemental materials.
Q5. Lines 125-126. Are there any duplicates on Table S1?
AU: No. It was typo in the main text and we changed 73,024 to 73,023.
Q6. Lines 132-133. Figure S3. The term "CpG islandmaked" is used in the table. Is this the correct term?
AU: We calculated methylation level of the CpG island after the repeat region was masked. To make it clear, we revised it to “CpG island” in the figure and described it in the legend.
Q7. Line 152. Figure S6. In the figure description, please define the X and Y axis.
AU: We added “The cattle assembly is represented as black bars with relative DNA methylation levels indicated by color lines above on the chromosomes.”
Q8. Lines 157-159. Please clarify the sentence beginning with "We evaluated..." You state that you "calculate the ratio between the observed density in sperm PMDs and the average density in autosomes". This is confusing because sperm contain autosomes. Please clarify the legend for Figure S7 as well.
AU: We changed “in sperm PMDs” to “in sperm-specific PMDs”.
Q9. Figure S8. Please define the vertical green and blue bars to the left of the image.
AU: We changed to “The 32 satellite-containing PMDs (labelled by blue bar on the left) showed lower methylation levels than the non-satellite-containing PMDs (labelled by green bar on the left) in sperm cells, which was not seen in the somatic cells.”
Q10. Line 173. When referring to the "satellite length divided by the region length", how is the region length defined? What parameters are used to define the region length?
AU: By the region length, we mean the PMD length. The PMD length was defined as described in Materials and methods.
Q11. Line 178. Chromosomal coordinates are listed for Figure 2a, right panel. Can you please list chromosomal coordinates for the left and middle panels as well?
AU: Left panel: chr29:1-560,000 and middle panel: chr29:30,220,001-30,400,000. We also added them in the main text.
Q12. Lines 205-206. The sentence beginning "In sperm, all PMD..." Can you please clarify this sentence?
AU: We changed it to “In sperm, all PMDs were supported by HMRs (with overlap counts ranging from 14 to 567), while only 13.5% HMRs were supported by the PMDs.”
Q13. Lines 215-217. The sentence beginning "On the other hand, the O/E..." Can you please clarify this sentence?
AU: It was changed into “On the other hand, the O/E value of the satellite regions, which overlap with somatic tissue-specific HMRs, was only 0.23.”
Q14. Line 221. Should Table S19 be Table S10?
AU: Yes, we changed to S10.
Q15. Line 224. Figure S10. In the Figure legend, please define the black and green.
AU: We added “Green: gene-term association positively reported; Black: gene-term association not reported yet.”
Q16. Line 238. Figure 4d right panel should be Figure 4d left panel.
AU: Yes, we changed to “left”.
Q17. Line 250. Figure S11 should be Figure S10. Starting with this line, it seems like all references to supplemental figures are misidentified. For example, lines 256, 276 through the end of the manuscript. Please double check that the correct supplemental figures are referenced.
AU: Sorry. We added the missing Figure S10 back. Now all supplemental figures are fine.
Q18. Lines 257-258. "Their average methylation levels were significantly higher in sperm cells than the somatic cells (Figure 4e)." Figure 4e shows the opposite.
AU: We changed “higher” to “lower”.
Q19. Line 275. The comment about sequence divergence was confusing until I read line 374. In line 275 or that section, please consider clarifying that sequence divergence is referring to divergence

from the consensus sequence.
AU: We added it.
Q20. Line 278. "LINE, SINE, LRT, DNA..." The LINE, SINE and LTR are all components of DNA. When you refer to DNA what exactly are you referring to?
AU: DNA here is referred to one class of repeats, i.e. DNA transposons.
Q21. Line 291. TFBS should be defined on line 291 instead of line 296.
AU: We did this change.
Q22. Line 407. Is the brain region that you are using the prefrontal cortex? If so, please consistently use the same terminology to consistently define this brain region, throughout the manuscript, figures, tables and supplemental information.
AU: Yes, it is. We fixed them throughout.
Q23. Sample Collection. In humans and mice, methylation brain tissues has been shown to be different than other somatic tissues. For example, there is a higher prevalence of hydroxymethylation in brain tissues compared to somatic tissues. The issue with this is that WGBS cannot distinguish 5-methylcytosine from 5-hydroxymethylcytosine. Therefore, your results have the potential to be somewhat skewed.
AU: Yes, we agree with this statement. 5hmC is beyond this study and will be studied in the future.
Q24. Line 407. Were the adult Holstein cows related?
AU: They are related via the same paternal grandsire.
Q25. Line 409. The authors state that semen straws were collected twice from two fertile bulls. For each animal, were both semen straws collected at the same time or at different time points?
AU: Two different times.
Q26. Line 419. Please specify if the barcodes used were from Illumina or another source.
AU: They were from Illumina.
Q27. Line 422. Was PCR used for amplification? If so how many cycles?
AU: Yes, 8-12 cycles.
Q28. Materials and Methods. Please specify the parameters that were used for each program, even if the default parameters were used. Also, please cite all programs used.
AU: As you requested, we added all necessary parameters for important pipelines.
Q29. Line 434. Please include the reference for UMD3.1 assembly.
AU: It was added.
Q30. Lines 443-444. The authors state the criteria used for identifying DMCs. How was this criteria defined?
AU: DMC detection were applied using a R package (methykit, R version 3.3.3). Please refer to Akalin A, Kormaksson M, Li S, Garrett-Bakelman FE, Figueroa ME, Melnick A, Mason CE. methylKit: a comprehensive R package for the analysis of genome-wide DNA methylation profiles. Genome Biol. 2012 Oct 3;13(10):R87.
Q31. Line 447. The authors state "as 1,000 bp around..." Please specify if this is ± 1,000 bp of the TSS.
AU: Yes, it is ± 1,000 bp of the TSS.
Q32. Line 451. "An in-house R script was used to plot the comparison results." Is the R-script publically available?
AU: This script use commonly used packages, such as plot(), to draw figures. We changed to "R packages were used to plot the comparison results."
Q33. Lines 455-457. "According to the distribution of the window's...65% and 70%." What additional criteria was used to identify 60% methylation as the threshold?
AU: This was based on the line plot of the PMD methylation level distribution (Figure S5A), and 60% was chosen as the threshold.
Q34. Line 459. "one R package" can you please provide a reference or website?
AU: HMM is the R package (https://cran.r-project.org/web/packages/HMM/).
Q35. Lines 466-467. Please clarify the sentence beginning: "The GenomicRanges package in R were

used to statistics..."
AU: It was changed to “The GenomicRanges package in R were used to calculate statistics of the overlapped HMRs in different tissues or sperms.”
Q36. Lines 479-481. "The STRING..." Please rephrase this sentence for clarity.
AU: We added “software” after STRING.
Q37. I searched GEO with the accession number provided to confirm data availability and my search yielded the following: "Accession "GSE106538" is currently private and is scheduled to be released on Nov 01, 2018."
AU: Once accepted, the accession will be made public.

--
Please also take a moment to check our website at https://giga.editorialmanager.com/l.asp?i=26738&l=FWLWAN8T for any additional comments that were saved as attachments. Please note that as GigaScience has a policy of open peer review, you will be able to see the names of the reviewers.

Close
