# Supplementary material for: Comparative whole genome DNA methylation profiling of cattle sperm and somatic tissues reveals striking hypomethylated patterns in sperm
Source: Gigascience. 2018 Apr 10;7(5):giy039. doi: 10.1093/gigascience/giy039 (PMC5928411; doi:10.1093/gigascience/giy039)
Supplement: Response_to_Reviewer_Comments_Revision_1.pdf [file giy039_response_to_reviewer_comments_revision_1.pdf]

Author's Response To Reviewer Comments

Close

Revision

- As suggested by the reviewer (see below), please explain and discuss the use of 5x coverage (and potential limitations connected to this) in more detail in the manuscript itself.
AU: In Discussion, On Line 402, we added a paragraph discussing these.

- Prior to acceptance, the GEO accessions need to be public. To help our data curators to assess the (meta)data, it will speed things up if you send us details for review access to the (currently private) GEO data, if you have them. (Actually, we also give the reviewers full data access - it seems I might have have failed to ask for the GEO review access earlier, at least I can't find any record of it at the moment?).
AU: GSE106538 was made public on March 8, 2018 and your curator has already gone through it at https://www.ncbi.nlm.nih.gov/geo/query/acc.cgi?acc=GSE106538

- Article type: You call your work a "comprehensive resource" and I wonder whether it may be better suited as a "Data Note" rather than a "Research Article"(https://academic.oup.com/gigascience/pages/data_note). However, a case can probably be made for both classifications, as you also show some analysis. Do you have strong preferences either way?
AU: We prefer Research Article.

For your information: Data notes are indexed the same way as Research Articles. The Article Processing Charge for a Data Note is much lower (see https://academic.oup.com/gigascience/pages/charges_licensing_and_self_archiving).

- Please structure your abstract accordingly (Data Note: "Background - Findings - Conclusions" / Research: "Background - Results - Conclusions").

- You show a large number of supplemental files that are mentioned in the text, which is good. I think it would help readers if you also add a list of the supplemental material at the end of the main manuscript, with a short description of the content of each supplemental file.
AU: They are added after Materials and methods.

- Our data curators will contact you regarding supporting data. We may prepare a GigaDB dataset that can be linked from the main paper, but my colleagues from the GigaDB team will discuss this with you.
AU: He contacted us with a few questions. We provided our answers already.

Close
