# Supplementary material for: Comparative whole genome DNA methylation profiling of cattle sperm and somatic tissues reveals striking hypomethylated patterns in sperm
Source: Gigascience. 2018 Apr 10;7(5):giy039. doi: 10.1093/gigascience/giy039 (PMC5928411; doi:10.1093/gigascience/giy039)
Supplement: Reviewer_1_Report_(Revision_1) -- Ole Madsen — 2/24/2018 Reviewed [file giy039_reviewer_1_report_(revision_1).pdf]

Reviewer Report

Title: Comparative whole genome DNA methylation profiling of cattle sperm and somatic tissues reveals striking hypomethylated patterns in sperm

Version: Revision 1 **Date:** 2/24/2018

Reviewer name: Ole Madsen

Reviewer Comments to Author:

Read the revised version of Zhou et al. I only have one mall comment.
1) Think the authors should include a (couple of) sentence(s) explaining their use of 5x for the regions.

Methods

Are the methods appropriate to the aims of the study, are they well described, and are necessary controls included? Yes

Conclusions

Are the conclusions adequately supported by the data shown? Yes

Reporting Standards

Does the manuscript adhere to the journal's guidelines on minimum standards of reporting? Yes

Choose an item.

Statistics

Are you able to assess all statistics in the manuscript, including the appropriateness of statistical tests used? Yes, and I have assessed the statistics in my report.

Quality of Written English

Please indicate the quality of language in the manuscript: Acceptable

Declaration of Competing Interests

Please complete a declaration of competing interests, considering the following questions:

- Have you in the past five years received reimbursements, fees, funding, or salary from an organisation that may in any way gain or lose financially from the publication of this manuscript, either now or in the future?

- Do you hold any stocks or shares in an organisation that may in any way gain or lose financially from the publication of this manuscript, either now or in the future?
- Do you hold or are you currently applying for any patents relating to the content of the manuscript?
- Have you received reimbursements, fees, funding, or salary from an organization that holds or has applied for patents relating to the content of the manuscript?
- Do you have any other financial competing interests?
- Do you have any non-financial competing interests in relation to this paper?

If you can answer no to all of the above, write 'I declare that I have no competing interests' below. If your reply is yes to any, please give details below.

I declare that I have no competing interests

I agree to the open peer review policy of the journal. I understand that my name will be included on my report to the authors and, if the manuscript is accepted for publication, my named report including any attachments I upload will be posted on the website along with the authors' responses. I agree for my report to be made available under an Open Access Creative Commons CC-BY license (http://creativecommons.org/licenses/by/4.0/). I understand that any comments which I do not wish to be included in my named report can be included as confidential comments to the editors, which will not be published.

I agree to the open peer review policy of the journal

To further support our reviewers, we have joined with Publons, where you can gain additional credit to further highlight your hard work (see: https://publons.com/journal/530/gigascience). On publication of this paper, your review will be automatically added to Publons, you can then choose whether or not to claim your Publons credit. I understand this statement.

Yes Choose an item.
