# Supplementary material for: Comparative whole genome DNA methylation profiling of cattle sperm and somatic tissues reveals striking hypomethylated patterns in sperm
Source: Gigascience. 2018 Apr 10;7(5):giy039. doi: 10.1093/gigascience/giy039 (PMC5928411; doi:10.1093/gigascience/giy039)
Supplement: Reviewer_2_Report_(Original_Submission) -- Stephanie McKay — 1/23/2018 Reviewed [file giy039_reviewer_2_report_(original_submission).pdf]

Reviewer Report

Title: Comparative whole genome DNA methylation profiling of cattle sperm and somatic tissues reveals striking hypomethylated patterns in sperm

Version: Original Submission **Date:** 1/23/2018

Reviewer name: Stephanie McKay

Reviewer Comments to Author:

1. There are minor grammar issues throughout the manuscript, please check the grammar.
2. Line 48. Brain is listed as a tissue, please define the precise brain region used in this study.
3. Line 122. The Figure description for Figure S1 contains track changes. Please correct the figure legend.
4. Line 124. You refer to Figure S2 and state "successfully separated brain". However, for Figure S2, brain is labeled as cortex. Please specify which brain cortex is used. Please make this change throughout the manuscript and supplemental materials.
5. Lines 125-126. Are there any duplicates on Table S1?
6. Lines 132-133. Figure S3. The term "CpG islandmaked" is used in the table. Is this the correct term?
7. Line 152. Figure S6. In the figure description, please define the X and Y axis.
8. Lines 157-159. Please clarify the sentence beginning with "We evaluated..." You state that you "calculate the ratio between the observed density in sperm PMDs and the average density in autosomes". This is confusing because sperm contain autosomes. Please clarify the legend for Figure S7 as well.
9. Figure S8. Please define the vertical green and blue bars to the left of the image.
10. Line 173. When referring to the "satellite length divided by the region length", how is the region length defined? What parameters are used to define the region length?
11. Line 178. Chromosomal coordinates are listed for Figure 2a, right panel. Can you please list chromosomal coordinates for the left and middle panels as well?
12. Lines 205-206. The sentence beginning "In sperm, all PMD..." Can you please clarify this sentence?
13. Lines 215-217. The sentence beginning "On the other hand, the O/E..." Can you please clarify this sentence?
14. Line 221. Should Table S19 be Table S10?
15. Line 224. Figure S10. In the Figure legend, please define the black and green.
16. Line 238. Figure 4d right panel should be Figure 4d left panel.
17. Line 250. Figure S11 should be Figure S10. Starting with this line, it seems like all references to supplemental figures are misidentified. For example, lines 256, 276 through the end of the manuscript. Please double check that the correct supplemental figures are referenced.
18. Lines 257-258. "Their average methylation levels were significantly higher in sperm cells than the somatic cells (Figure 4e)." Figure 4e shows the opposite.
19. Line 275. The comment about sequence divergence was confusing until I read line 374. In line 275 or that section, please consider clarifying that sequence divergence is referring to divergence from the consensus sequence.
20. Line 278. "LINE, SINE, LRT, DNA..." The LINE, SINE and LTR are all components of DNA. When you refer to DNA what exactly are you referring to?
21. Line 291. TFBS should be defined on line 291 instead of line 296.
22. Line 407. Is the brain region that you are using the prefrontal cortex? If so, please consistently use the same terminology to consistently define this brain region, throughout the manuscript, figures, tables and supplemental information.
23. Sample Collection. In humans and mice, methylation brain tissues has been shown to be different than other somatic tissues. For example, there is a higher prevalence of hydroxymethylation in brain tissues compared to somatic tissues. The issue with this is that WGBS cannot distinguish 5-methylcytosine from 5-

hydroxymethylcytosine. Therefore, your results have the potential to be somewhat skewed.
24. Line 407. Were the adult Holstein cows related?
25. Line 409. The authors state that semen straws were collected twice from two fertile bulls. For each animal, were both semen straws collected at the same time or at different time points?
26. Line 419. Please specify if the barcodes used were from Illumina or another source.
27. Line 422. Was PCR used for amplification? If so how many cycles?
28. Materials and Methods. Please specify the parameters that were used for each program, even if the default parameters were used. Also, please cite all programs used.
29. Line 434. Please include the reference for UMD3.1 assembly.
30. Lines 443-444. The authors state the criteria used for identifying DMCs. How was this criteria defined?
31. Line 447. The authors state "as 1,000 bp around…" Please specify if this is ± 1,000 bp of the TSS.
32. Line 451. "An in-house R script was used to plot the comparison results." Is the R-script publically available?
33. Lines 455-457. "According to the distribution of the window's…65% and 70%." What additional criteria was used to identify 60% methylation as the threshold?
34. Line 459. "one R package" can you please provide a reference or website?
35. Lines 466-467. Please clarify the sentence beginning: "The GenomicRanges package in R were used to statistics…"
36. Lines 479-481. "The STRING…" Please rephrase this sentence for clarity.
37. I searched GEO with the accession number provided to confirm data availability and my search yielded the following: "Accession "GSE106538" is currently private and is scheduled to be released on Nov 01, 2018."

Methods

Are the methods appropriate to the aims of the study, are they well described, and are necessary controls included? Yes

Conclusions

Are the conclusions adequately supported by the data shown? Yes

Reporting Standards

Does the manuscript adhere to the journal's guidelines on minimum standards of reporting? Yes

Choose an item.

Statistics

Are you able to assess all statistics in the manuscript, including the appropriateness of statistical tests used? Yes, and I have assessed the statistics in my report.

Quality of Written English

Please indicate the quality of language in the manuscript: Needs some language corrections before being published

Declaration of Competing Interests

Please complete a declaration of competing interests, considering the following questions:

- Have you in the past five years received reimbursements, fees, funding, or salary from an organisation that may in any way gain or lose financially from the publication of this manuscript, either now or in the future?
- Do you hold any stocks or shares in an organisation that may in any way gain or lose financially from the publication of this manuscript, either now or in the future?
- Do you hold or are you currently applying for any patents relating to the content of the manuscript?
- Have you received reimbursements, fees, funding, or salary from an organization that holds or has applied for patents relating to the content of the manuscript?
- Do you have any other financial competing interests?
- Do you have any non-financial competing interests in relation to this paper?

If you can answer no to all of the above, write 'I declare that I have no competing interests' below. If your reply is yes to any, please give details below.

I declare that I have no competing interests

I agree to the open peer review policy of the journal. I understand that my name will be included on my report to the authors and, if the manuscript is accepted for publication, my named report including any attachments I upload will be posted on the website along with the authors' responses. I agree for my report to be made available under an Open Access Creative Commons CC-BY license (http://creativecommons.org/licenses/by/4.0/). I understand that any comments which I do not wish to be included in my named report can be included as confidential comments to the editors, which will not be published.

I agree to the open peer review policy of the journal

To further support our reviewers, we have joined with Publons, where you can gain additional credit to further highlight your hard work (see: https://publons.com/journal/530/gigascience). On publication of this paper, your review will be automatically added to Publons, you can then choose whether or not to claim your Publons credit. I understand this statement.

Yes Choose an item.
